# Self-Assembled Materials Based on Fully Aromatic Peptides: The Impact of Tryptophan, Tyrosine, and Dopa Residues

**DOI:** 10.1021/acs.langmuir.3c03214

**Published:** 2024-01-04

**Authors:** Nicole Balasco, Davide Altamura, Pasqualina Liana Scognamiglio, Teresa Sibillano, Cinzia Giannini, Giancarlo Morelli, Luigi Vitagliano, Antonella Accardo, Carlo Diaferia

**Affiliations:** † Institute of Molecular Biology and Pathology, CNR, Piazzale Aldo Moro 5, Rome 00185, Italy; ‡ Institute of Crystallography (IC), CNR, Via Amendola 122, Bari 70126, Italy; § Department of Sciences, University of Basilicata, Via dell’Ateneo Lucano 10, Potenza 85100, Italy; ∥ Department of Pharmacy and CIRPeB, Research Centre on Bioactive Peptides “Carlo Pedone”, University of Naples “Federico II”, Via Montesano 49, Naples 80131, Italy; ⊥ Institute of Biostructures and Bioimaging (IBB), CNR, Via Castellino 111, Naples 80131, Italy

## Abstract

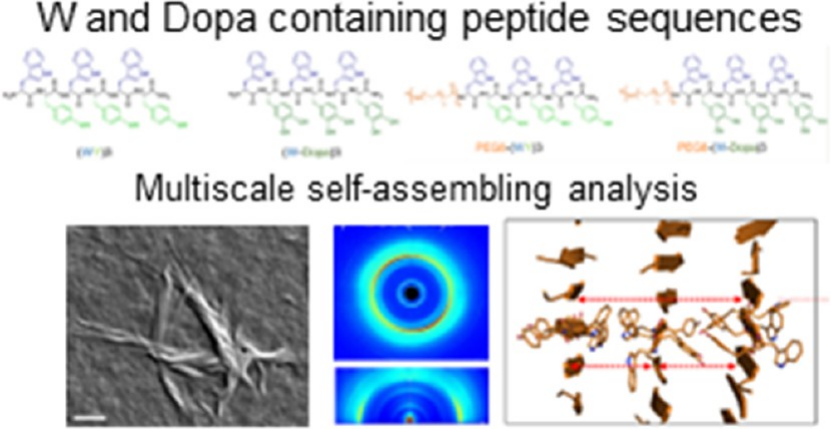

Peptides are able to self-organize in structural elements including cross-β structures. Taking advantage of this tendency, in the last decades, peptides have been scrutinized as molecular elements for the development of multivalent supramolecular architectures. In this context, different classes of peptides, also with completely aromatic sequences, were proposed. Our previous studies highlighted that the (FY)3 peptide, which alternates hydrophobic phenylalanine and more hydrophilic tyrosine residues, is able to self-assemble, thanks to the formation of both polar and apolar interfaces. It was observed that the replacement of Phe and Tyr residues with other noncoded aromatic amino acids like 2-naphthylalanine (Nal) and Dopa affects the interactions among peptides with consequences on the supramolecular organization. Herein, we have investigated the self-assembling behavior of two novel (FY)3 analogues with Trp and Dopa residues in place of the Phe and Tyr ones, respectively. Additionally, PEGylation of the N-terminus was analyzed too. The supramolecular organization, morphology, and capability to gel were evaluated using complementary techniques, including fluorescence, Fourier transform infrared spectroscopy, and scanning electron microscopy. Structural periodicities along and perpendicular to the fiber axis were detected by grazing incidence wide-angle X-ray scattering. Finally, molecular dynamics studies provided interesting insights into the atomic structure of the cross-β that constitutes the basic motif of the assemblies formed by these novel peptide systems.

## Introduction

**Figure 1:**
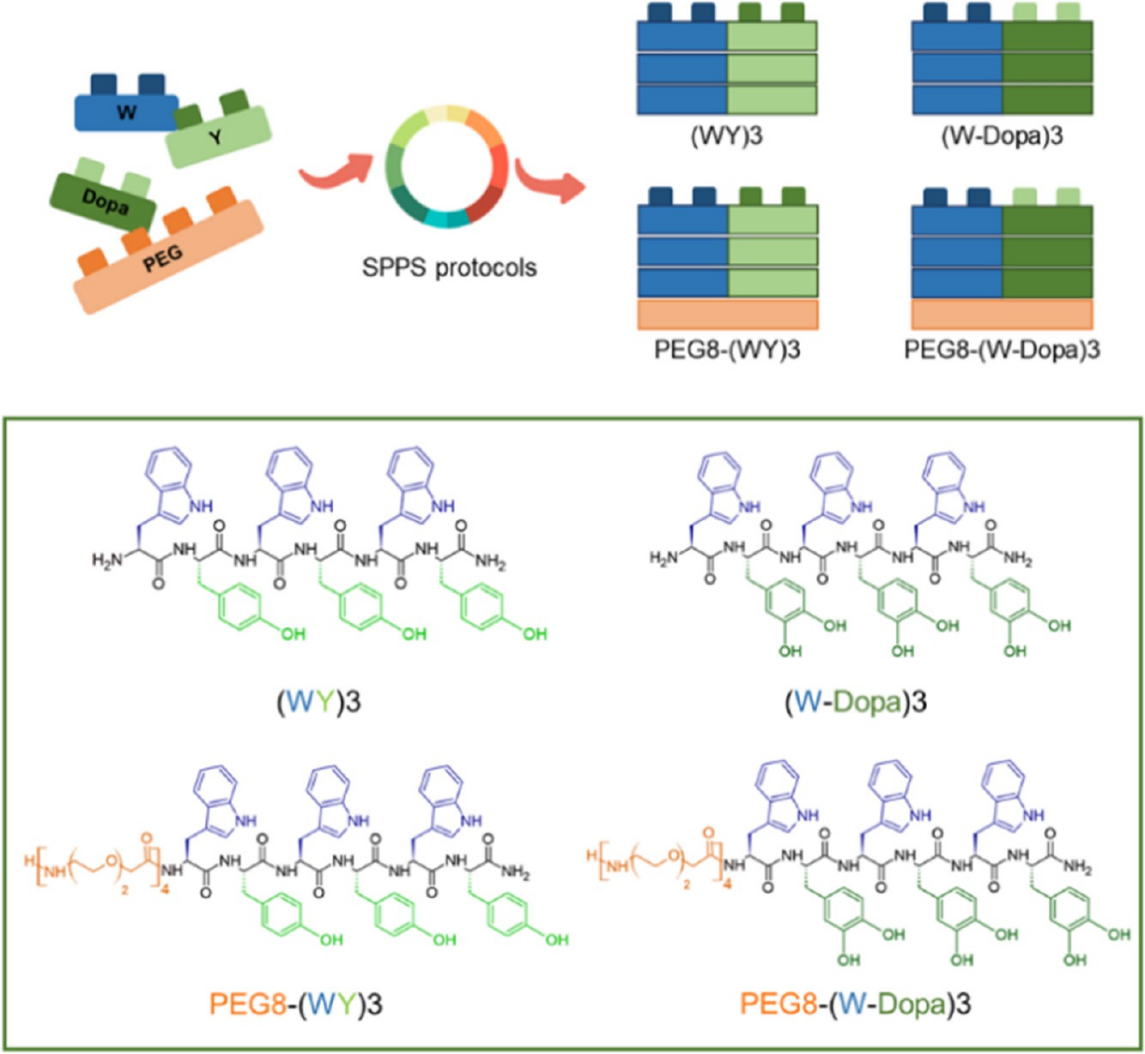
Schematic representation of (WY)3, (W-Dopa)3, and the corresponding PEGylated peptides PEG8-(WY)3 and PEG8-(W-Dopa)3.

Nanostructures and nanomaterials recently emerged as smart platforms for biomedical and technological applications.^1−13^ Their main advantage is related to the possibility of simultaneously performing different tasks like diagnosis and therapy in theranostic systems or tissue regeneration, imaging, and delivery of therapeutic molecules in extracellular matrices. Most of the nanostructures are formulated starting from commercial and synthetic polymers, surfactants, or phospholipids. However, the research is also focused on the exploitation of novel suitable building blocks, which can allow the overcoming of some limitations of the traditional ones. Among them, short and ultrashort peptide sequences have been envisioned and investigated for the fabrication of innovative nanomaterials like fibers, nanotubes, nanospheres, and hydrogels.^14−18^ These supramolecular architectures can be obtained by taking advantage of noncovalent interactions that are established between the side chains of amino acid residues or between the head–tail portions of the peptide backbone. Hydrogen bonds, van der Waals interactions, and aromatic π–π stacking are considered as the most relevant forces able to promote the self-aggregation of peptide sequences. The choice and the design of building blocks can deeply affect the entity and the nature of their mutual interactions and, in turn, the structural and functional properties of the final material. In this context, the importance of developing novel peptide sequences to modulate and improve the performance of supramolecular nanosystems clearly emerges. Taking inspiration from the impressive literature about nanostructures based on diphenylalanine (FF) homodimer self-assembling,^19−23^ we recently described fibers and hydrogels based on short aromatic peptides.^24−27^ The ancestor peptide PEG8-F6^24^ and its analogues (PEG12-F6, PEG18-F6, and PEG24-F6),^25^ containing a hexaphenylalanine derivatized with a polyethylene glycol (PEG) moiety at its N-terminus, self-assemble in water into ordered fibers. On the contrary, the PEG8-(FY)3 analogue,^26^ in which three Phe residues have been replaced with three Tyr ones, forms soft, self-supporting hydrogels. Molecular modeling and dynamics simulations on assemblies demonstrated that the alternation of hydrophobic and hydrophilic aromatic amino acid residues allowed the formation of two distinct interfaces: an apolar one, made by facing Phe residues, and a polar one constituted by Tyr side chains of facing strands. Successively, we also reported the synthesis of three novel analogues [(Nal-Y)3, (F-Dopa)3, and (Nal-Dopa)3], in which the natural amino acids (Phe and Tyr) of the (FY)3 hexapeptide were replaced with the noncoded ones, 2-naphthylalanine (Nal) and 3,4-dihydroxy-l-phenylalanine (Dopa).^27^ The effect of PEGylation of the N-terminus of these peptides was also evaluated. Structural and rheological characterization highlighted that all the non-PEGylated peptides and PEG8-(Nal-Y)3 were able to form self-supporting soft gels at 1.0 wt %, exhibiting the following stiffness scale: *G*′(Nal-Dopa)3 > *G*′(Nal-Y)3 > *G*′PEG8-(Nal-Dopa)3 > *G*′(F-Dopa)3. Naphthylalanine was chosen in place of phenylalanine, having a more extended aromatic group in its side chain. Herein, we report the synthesis and structural characterization of other aromatic (FY)3 analogues [viz., (WY)3, (W-Dopa)3, PEG8-(WY)3, and PEG8-(W-Dopa)3] obtained by replacing the Phe residue with the more sterically hindered and more hydrophilic tryptophan (Figure 1).

The expected low solubility of these peptides was compensated for by the monodisperse PEGylation of the N-terminus. The secondary structure, morphology, and rheological properties of all the designed peptides were assessed by a set of techniques including fluorescence, Fourier transform infrared (FT-IR) spectroscopy, scanning electron microscopy (SEM), and wide-angle X-ray scattering in grazing incidence (GIWAXS) and transmission (WAXS). Finally, molecular dynamics (MD) studies have provided interesting insights into the atomic structure, stability in terms of organization and self-structuration, and the cross-β that constitutes the basic motif of the assemblies formed by these novel peptide systems.

## Experimental Section

### Chemicals and Instrumentations

N^α^-Fmoc-amino-protected acid, Rink amide MBHA (4-methylbenzhydrylamine) resin, and coupling reagents are commercially available from Merck (Milan, Italy). The N^α^-Fmoc-Dopa(acetonide)-OH is reachable from Iris Biotech GMBH (Marktredwitz, Germany). The monodisperse Fmoc-8-amino-3,6-dioxaoctanoic acid Fmoc-AdOO-OH (PEG2) was purchased from Neosystem (Strasbourg, France). All other chemicals are commercially available from Merck or Carlo Erba (Cornaredo, Italy). All the products were used as delivered. Peptide solutions were prepared by weight using double distilled water. Preparative RP-HPLC was carried out using an LC8 Shimadzu HPLC system (Shimadzu Corporation, Kyoto, Japan) equipped with a UV lambda-Max Model 481detector. A Phenomenex (Torrance, CA) C_18_ column was selected as the stationary phase. The elution phase was composed of H_2_O/0.1% TFA (line A) and CH_3_CN/0.1% TFA (line B). The gradient was 20 to 70% over 30 min at a flow rate of 20 mL/min. The purity and the identity of the products were assessed by analytical LC-MS analysis using a Finnigan Surveyor MSQ single quadrupole electrospray ionization spectrometer (Finnigan/Thermo Electron Corporation, San Jose, CA) with a C_18_-Phenomenex column eluting with H_2_O/0.1% TFA (A) and CH_3_CN/0.1% TFA (B) from 20 to 80% over 20 min at a flow rate of 200 μL/min.

### Solid-Phase Peptide Synthesis (SPPS)

Peptide sequences were synthesized according to standard SPPS (solid-phase peptide synthesis) procedures with a Fmoc/tBu chemistry, as previously described.^24−27^ Briefly, synthesis (scale = 0.30·10^–3^ mol) was carried out on Rink amide MBHA resin (substitution 0.71 mmol/g) using a DMF/NMP (1/1, v/v) mixture as solvent. N^α^-Fmoc deprotection was achieved via two treatments with 30% (v/v) piperidine in DMF/NMP for 8 min each. Each amino acid coupling was performed twice for 35 min using a 2-fold molar excess of the protected Fmoc-amino acid, mixed with equimolar amounts of 1-hydroxybenzotriazole (HOBt), *N*,*N*,*N*′,*N*′-tetramethyl-*O*-(1*H*-benzotriazol-1-yl)uronium hexafluorophosphate (HBTU), and a 4-fold molar excess of diisopropylethylamine (DIPEA). Fmoc-AdOO-OH (PEG2) were sequentially coupled in the solid phase, as previously described.^24−27^ Crude peptides were fully cleaved from the resin with a TFA (trifluoroacetic acid)/TIS (triisopropylsilane)/H_2_O (92.5/5/2.5, v/v/v) mixture at room temperature for 3 h. Peptides were precipitated with ice-cold water and freeze-dried three times. The purification of the crude products was carried out by RP-HPLC. The mass spectra confirmed the identity of the product. To remove putative residual TFA, after three freeze-drying cycles, each powder was again lyophilized three times from solutions in which 0.01 mol/L HCl was added to favor the anion exchange.

### Preparation of Peptide Solutions and Hydrogels

Peptide solutions were prepared by direct dissolution of pure powders in bidistilled water. The analytical concentration of solutions was spectroscopically determined by absorbance on a UV–vis Thermo Fisher Scientific Inc. (Wilmington, Delaware, USA) Nanodrop 2000c spectrophotometer equipped with a 1.0 cm quartz cuvette (Hellma) using the molar absorptivity (ε) at 280 nm reported in Table 1. For each peptide, the ε was calculated using the formula ε_(280)_= [*x*·5500 + *y*·1215 + *k*·2630] [L·cm^–1^·mol^–1^], where *x*, *y*, and *k* were the numbers of W, Y, and Dopa residues, respectively. The solubility values analytically determined were 1.15, 3.77, 7.61, and 15.00 mg/mL for (WY)3, PEG8-(WY)3, (W-Dopa)3, and PEG8-(W-Dopa)3, respectively. Hydrogel formation was triggered using the “solvent switch” method, which consists of the addition of water to a peptide stock solution (100 mg/mL in DMSO). The peptide concentration after water addition was 1.0 wt % (10.0 mg/mL).

**Table 1:** Formula, Theoretical and Experimentally Found Molecular Weight (MW), and Molar Absorptivity of the Investigated Polyaromatic Peptides

**compound**	**formula**	**M**W_**calc.**_**(a.m.u.)**	**MW**_**deter**_**(a.m.u.)**	**ε**_**280 nm**_
(WY)3	C_60_H_60_N_10_O_9_	1065.2	1065.6	20,145
PEG8-(WY)3	C_84_H_104_N_14_O_21_	1645.8	1646.8	20,145
(W-Dopa)3	C_60_H_60_N_10_O_12_	1113.2	1113.5	24,390
PEG8-(W-Dopa)3	C_84_H_104_N_14_O_24_	1693.8	1694.7	24,390

### Fluorescence Study and Determination of Critical Aggregation Concentration

The fluorescence spectra of peptide solution were collected at room temperature on a Jasco spectrofluorophotometer (model FP-750), exciting the samples as λ_ex_ = 280 nm with a voltage of 700 V. The determination of the critical aggregation concentration (CAC) value was achieved for all the W-containing sequences by fluorescence titration of the dye 8-anilino-1-naphthalene sulfonic acid ammonium salt (ANS) with increasing amounts of the peptide solution.^28^ All the spectra, acquired in a quartz cell with a 1.0 cm path length at room temperature, were recorded using the following settings: excitation and emission bandwidths = 5 nm, recording speed = 120 nm·min^–1^, excitation wavelength = 350 nm, and automatic selection of the time constant. The measurement was performed by adding small aliquots of peptide derivatives in 200 μL of a 20 μmol·L^–1^ ANS water solution. At the end of the titration, the blank was subtracted. The fluorescence spectra were corrected for the blank and adjusted for the dilution. Tendency lines were extracted using a least-squares method extrapolation. Titrations were conducted in duplicate.

### FT-IR Spectroscopy

The FT-IR spectra of all peptide solutions at their maximum concentration were collected on a Jasco FT/IR 4100 spectrometer (Easton, MD) in an attenuated total reflection (ATR) mode and using a Ge single crystal at a resolution of 4 cm^–1^. A total of 250 scans of each peptide solution were recorded at a rate of 2 mm·s^–1^ and against a background of KBr. After collection in a transmission mode, the spectra were converted to emission. A quantitative multivariate analysis for secondary structure estimation (SSE) using a method of principal component regression (PCR) was conducted using a Jasco SSE dedicated software.

### Thioflavin T Spectroscopic Assay at the Solid State

W-containing peptides underwent a thioflavin T (ThT) assay at the solid state. Solution (20 μL) of all the peptides at their maximum concentration was dried under vacuum for 24 h. The dry peptide films were stained for 5 min with 15 μL of a water solution of 50 μmol·L^–1^ ThT. After removing the dye excess with a filter paper, the samples were dried overnight. Fluorescence images of the dried samples were recorded using a fluorescence microscope. Images were taken with a Leica DFC320 video camera (Leica, Milan, Italy) connected to a Leica DMRB microscope equipped with a 20× objective and green fluorescent protein (GPF) filter. The software ImageJ (National Institutes of Health, Bethesda, MD) was used for analysis.

### Congo Red Assay

The UV/vis Congo red (CR) spectroscopy assay was carried out using a freshly prepared stock solution of CR (1.75 mg·mL^–1^) in water, filtered through a 0.2 μm syringe immediately before its use. A small aliquot (2 μL) of this solution was added to 400 μL of W-peptide-containing solutions at their maximum solubility. After an incubation time of 15 min, the UV/vis spectra were recorded in a 1 cm quartz cell. The background of the dye was subtracted using a CR spectrum in water as a reference solution.

### SEM

SEM samples were prepared from a solution of aggregates at the maximum concentration. Approximately 10 μL of each sample was placed on a glass coverslip and left to dry under ambient conditions. The dried samples were coated with Au for conductance and viewed by using a scanning electron microscope (JEOL, Tokyo, Japan) operating at 10 kV.

### Wide-Angle X-ray Scattering (WAXS)/Grazing Incidence Wide-Angle Scattering (GIWAXS)

Fibers for WAXS analysis were prepared according to the stretch frame method using the same peptide solutions previously analyzed.^29^ Peptide solutions, at the maximum solubility, were also deposited on silicon substrates for GIWAXS experiments; a 0.18° incidence angle (below the critical angle for total reflection) was chosen to avoid parasitic scattering from the substrate. The fiber diffraction patterns were collected at the X-ray MicroImaging Laboratory (XMI-L@b) using a setup equipped with a Fr-E+ SuperBright rotating copper anode microsource (45 kV/55 mA; Cu *K*_α_, λ = 0.15405 nm, 2475 W) coupled through multilayer focusing optics (Confocal Max-Flux; CMF 15-105) to a three-pinhole camera (Rigaku SMAX3000). For WAXS and GIWAXS data collection, a 250 × 160 mm^2^ image-plate (IP) detector, with a 100 μm effective pixel size, was inserted at ∼10 or 8.7 cm downstream the sample, respectively, and read by an offline RAXIA reader.^30,31^

### Rheological Studies and Swelling Test

The rheological properties of gels were evaluated with a rotational controlled stress rheometer (Malvern Kinexus) using a 15 mm flat-plate geometry (PU20:PL61). A freshly prepared hydrogel sample (400 μL) at a concentration of 1.0 wt % was used. Each experiment was performed twice at 25 °C by using a humidity chamber and a gap of 1 mm. Preliminary dynamic rheological tests were carried out to identify the regime of linear viscoelasticity. The viscous elastic region was determined by oscillatory frequency (0.1–100 Hz) and strain sweep (0.01–100%). Then, a time-sweep oscillatory evaluation test (using a constant 0.1% strain and 1.0 Hz frequency) was performed for 20 min. The results are reported in Pascal (Pa) as the shear storage or elastic modulus (*G*′) and the shear loss or viscous modulus (*G*″). The swelling ratio was measured in triplicate by adding 1.5 mL of double distilled water on the top of each sample (1.0 wt %, *V* = 400 μL), followed by overnight incubation at 28 °C. Fully swollen hydrogels were weighed (*W*_s_) immediately after the removal of excess water. Samples were freeze-dried and weighed again (*W*_d_). The swelling behavior was expressed, according to eq 1, as the swelling ratio %*q*, which is the ratio between the weight of the swollen sample (*W*_s_) and the weight of the freeze-dried hydrogel (*W*_d_):
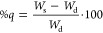
1

### Molecular Modeling: Systems and Notations

A three-dimensional model of (WY)3 aggregates was generated by applying the procedure previously reported.^24,26,32,33^ In particular, a model of (WY)3 composed of a single β-sheet made of 50 antiparallel β-strands (denoted as WY_ST50_SH1) was generated using the structure of the hexapeptide fragment KLVFFA of the amyloid-beta peptide II (PDB entry 3OW9) as the template.^34^ Two steric zipper models were then produced through the association of two of these sheets. However, to avoid any bias in the association modes of the sheets, in the starting models, they were well separated with a C^α^–C^α^ distance of approximately 16 Å. These two-sheet models were built by locating either the Trp (WY_ST50_SH2_WW) or Tyr (WY_ST50_SH_YY) side chains at the dry interface. Thus, Tyr or Trp residues were solvent exposed in WY_ST50_SH2_WW and WY_ST50_SH_YY, respectively. A more complex system was generated by considering three fifty-stranded β-sheets (WY_ST50_SH3). This assembly is endowed with two different steric zipper interfaces: one composed of Trp side chains and the other made of Tyr side chains. In this latter case, two surfaces made by either Trp or Tyr residues are solvent exposed.

### MD Protocol

The GROMACS software package 2020.3^35^ was used to carry out MD simulations on the (WY)3 models generated by molecular modeling. The Amber03 force field and the TIP3P water model were used. The systems were solvated with water molecules in triclinic boxes, and Cl^–^ counterions were added to balance charges (Table S1). Periodic boundary conditions were applied to the simulations. The systems were first energy minimized using the steepest descent (50,000 steps) and then equilibrated at a 300 K temperature for 500 ps (NVT) and a 1 atm pressure for 500 ps (NpT). The Velocity Rescaling and Parrinello–Rahman algorithms were used to control the temperature and pressure, respectively. Electrostatic interactions were computed using the particle-mesh Ewald (PME) method with a 1.2 Å grid spacing and a 10^–6^ relative tolerance. A 10 Å cutoff was used for the Lennard-Jones interactions. The LINCS algorithm was applied to constrain bond lengths. MD runs (200 ns time scale) were carried out at a constant temperature (300 K) and a constant pressure (1 atm) with a time step of 2 fs. The analysis of trajectory structures was performed using the VMD program^36^ and GROMACS routines.^35^

## Results and Discussion

### Peptide Design

Many different classes of low-molecular-weight peptides able to gel have not been reported in the literature until now. Among them, the PEG8-(FY)3 peptide, in which Phe and Tyr residues are alternated and derivatized with a PEG moiety, was the first example of a completely aromatic polymer–peptide conjugate hydrogelator.^26^ The gel formation in the PEG8-(FY)3 solution was explained as the consequence of the peculiar fibrillar supramolecular organization of the polymer–peptide building blocks in two chemically different interfaces endowed with either polar or apolar features. The apolar interface allocates all the Phe side chains, interacting via π–π stacking. On the contrary, the polar one contains Tyr phenolic groups. PEGylation on the hexapeptide was found to be essential for the gel formation, probably because it allows the achievement of an optimal balance between the hydrophilic/hydrophobic portions. Additionally, derivatization of the peptide with a monodisperse PEG moiety permits increasing the water solubility of peptides, without the insertion of resident charges that may compete with the self-assembling process.

It has been observed that the replacement of residues involved in the formation of one or both the interfaces can affect the supramolecular behavior and the gel formation properties,^27^ increasing the physical entrapment and retention capability of water and hence the mechanical stiffness of hydrogels.^27^ To deeply investigate the molecular determinant that can positively or negatively affect the aggregation process, herein, we evaluated the impact generated by replacement of the Phe residue with the Trp (W) one. Trp was selected to assess the effect of a more sterically hindered residue containing the heteroaromatic indole group. On the other side, Tyr residues were replaced by Dopa, characterized by the catechol group in place of the phenol one. Using this design, two novel peptides [(WY)3 and (W-Dopa)3] and their N-PEGylated analogues [PEG8-(WY)3 and PEG8-(W-Dopa)3] were designed (Figure 1). The novel sequences were synthesized using a solid-phase approach, according to the Fmoc/tBu protocols on Rink amide MBHA resin. This support allows for obtaining amidation of the C-terminus. Due to the major steric hindrance of the Trp residue, the synthesis of these primary sequences was more difficult than the previously reported ones and required the employment of a DMF/NMP mixture as solvent. After purification, peptides were lyophilized and their identity was assessed by LC-MS (Figures S1–S4 and Table 1). The estimation of the peptide solubility was spectroscopically determined by recording the absorbance spectra (Figure S5) on the centrifuged peptide solutions. The quantification of the peptide concentration pointed out the different solubility (1.5 mg/mL < S < 15.0 mg/mL) of the four peptides in water. As expected, peptides derivatized with the PEG moiety are more soluble than their corresponding unPEGylated versions, thus confirming PEGylation as a helpful strategy to improve intrinsic water solubility of all-aromatic sequences.

### Spectroscopic Characterization and CAC Determination

Peptide solutions at different concentrations were initially studied by fluorescence spectroscopy (Figure S6). Samples were excited at 280 nm, which corresponds to the absorption wavelengths of the aromatic residues (tryptophan, tyrosine, and Dopa). Differently from the expectation of the involvement of Trp in the formation of hydrophobic interactions, the fluorescence spectra of all the peptide solutions show an emission peak located between 350 and 360 nm, which indicates that Trp is surrounded by polar/aqueous solvent.^37^ This finding suggests that at the studied concentrations, peptides are in their monomeric form. In this perspective, the eventual occurrence of excimer species in solution was then investigated. From the inspection of the spectra reported in Figure S6, it can be concluded that only (WY)3 is prone to excimer formation in the studied concentration range.

**Figure 2:**
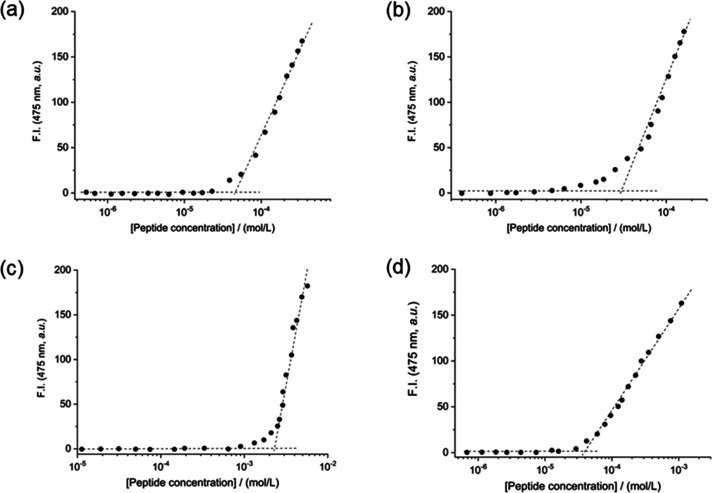
Fluorescence intensity of the ANS dye at 475 nm versus the concentration of (a) (WY)3, (b) PEG8-(WY)3, (c) (W-Dopa)3, and (d) PEG8-(W-Dopa)3 peptides. The CAC values are deduced from the curve break points.

Further information about the aggregation properties of W-containing peptides was obtained from the experimental determination of the CAC. The CAC values were estimated by the titration of a fluorophore, 8-anilinonaphthalene-1-sulfonate ammonium salt (ANS), with an increasing amount of peptide.^27^ By plotting the ANS fluorescence emission at 475 nm as a function of the peptide concentration, two tendency lines are identified using a least-squares method extrapolation approach. The CAC value is deduced in their intersection break point (Figure 2). The CAC values, collected in Table 2, point out the different propensity of each peptide to self-assemble under these experimental conditions. The (W-Dopa)3 derivative self-assembles into supramolecular structures above a CAC value of 2.3 × 10^–3^ mol/L. Unexpectedly, this value is 2 orders of magnitude higher than those exhibited by the other peptide derivatives (∼10^–5^ mol/L). These values do not seem to be affected by the log*P* values theoretically estimated by the ACD/3D Viewer and reported in Table 2. Probably, the higher CAC value of (W-Dopa)3 can be attributed to a multiparametric effect, taking into account the presence of two highly hindered residues, the absence of the PEG moiety, a different hydrophobic/hydrophilic balance, and both solvation and hydrophobic collapse effects.

**Table 2:** CAC Values (Expressed in mol/L and in μg/mL) of Peptide Derivatives and Their log*P* Values Theoretically Estimated by the ACD/3D Viewer

**compound**	**CAC** (mol/L)	**CAC** (μg/mL)	**log*P***
(WY)3	4.65 × 10^–5^	49.5	5.67 ± 0.90
PEG8-(WY)3	2.97 × 10^–5^	48.8	1.97 ± 1.03
(W-Dopa)3	2.30 × 10^–3^	2560	3.86 ± 0.90
PEG8-(W-Dopa)3	3.80 × 10^–5^	64.3	0.17 ± 1.03

### Structural Characterization in Solution

**Figure 3:**
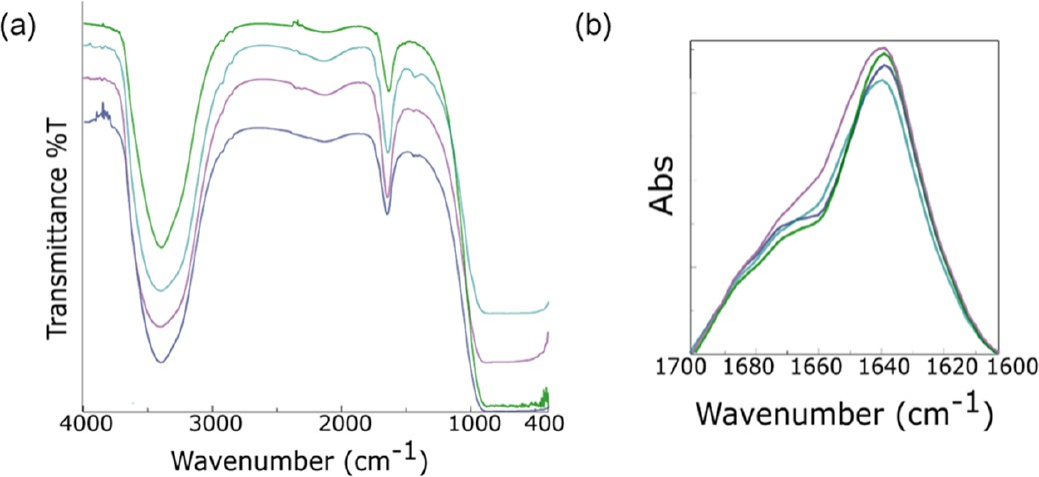
FT-IR analysis of the W-containing peptides. Transmittance spectra (a) and amide I absorbance deconvolution (b) for (WY)3 (light blue), PEG8-(WY)3 (green), (W-Dopa)3 (violet), and PEG8-(W-Dopa)3 (blue).

**Figure 4:**
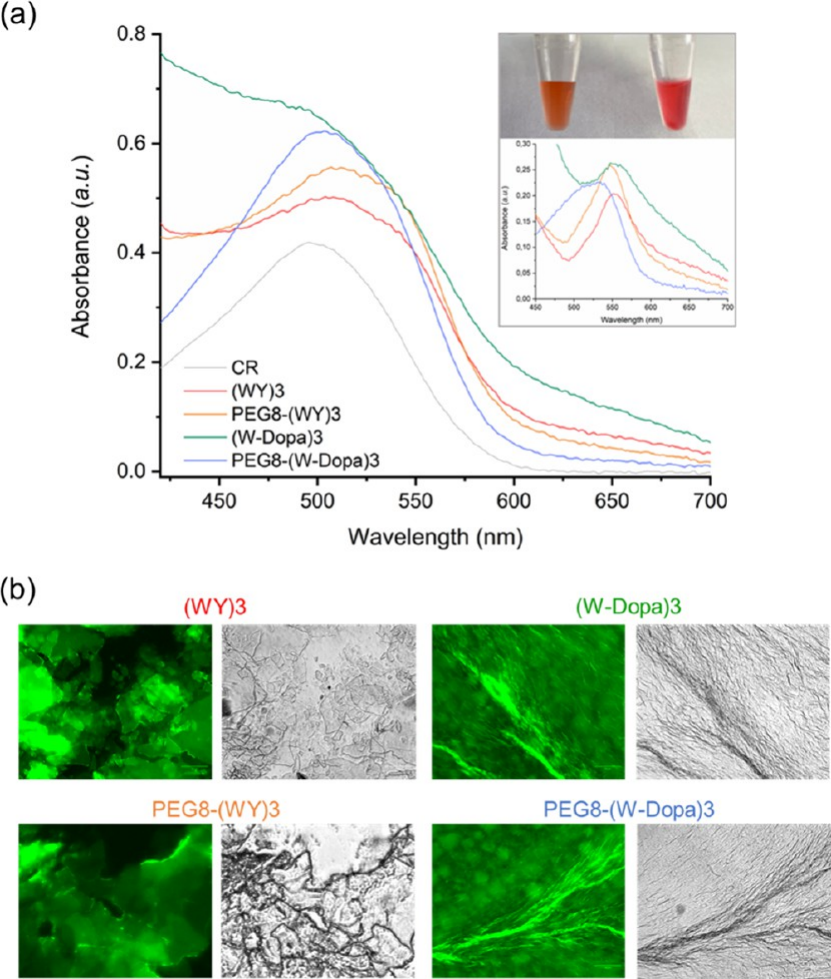
CR and ThT assays. (a) Absorbance spectra of CR alone or coincubated with peptide solutions. The subtraction of spectra and the macroscopical appearance of analyzed peptide solution alone or coincubated with CR are also reported in the inset. (b) Fluorescence and optical images of peptide solutions drop-casted on a glass slide, air-dried, and stained with the ThT solution. Samples are imaged in the spectral regions of the GFP (green fluorescent protein, λ_exc_ = 488 nm, λ_em_ = 507 nm) and in the bright field. Scale bar: 50 μm.

An FT-IR analysis allowed us to study the secondary structures adopted by W-containing peptides. The FT-IR spectra of peptide solutions, prepared by dissolving each peptide at its maximum solubility, share a common signature (Figure 3a) characterized by an intense transmittance signal between 3000 and 3700 cm^–1^ in the amide A region and a broad signal around 1640 cm^–1^ in the amide I region (1600–1700 cm^–1^). The signal in the amide A region can be attributed to the exposure of the aggregates to water, and it is generated by both symmetric and asymmetric stretching of O–H and N–H functional groups. Unfortunately, a deconvolution of this region, conducted to evaluate the contributions of N–H involved in H–bond interactions compared to the free N–H, did not permit the discrimination of one contributor from the other. On the other hand, the signal at 1640 cm^–1^ is associated with carbonyl stretching modes and it is reported to be secondary structure sensitive and related to the presence of β-rich assemblies.^38,39^ To reinforce and assess the band value, a first (*x*) derivative analysis was performed (Figure S7). We observed (*x*) = 0 at 1639 cm^–1^, thus indicating a common maximum for all of the spectra. For this, amide I deconvolution profiles in absorbance were also collected (Figure 3b). The prevalence of β-sheet structuration is reinforced by the presence of a major band at ∼1640 cm^–1^ for all of the samples, attributable to C=O stretching. The deconvolution analysis as weighted percentages of each secondary structure (Table S2) reinforces the previous finding about the prevalence of β-rich structuration. Moreover, the additional weak band at ∼1680–1690 cm^–1^ suggests the antiparallel orientation of the β-strands in assemblies. Notably, the trifluoroacetic acid,^40^ used as reactive for peptide cleavage and the chromatographic purification, may contribute to the amide I signal. However, the possibility of a residual TFA involvement in the signal can be excluded, considering that each peptide, after three lyophilization cycles, was additionally freeze-dried three times after a HCl treatment for TFA exchange. Additionally, the chemical nature of primary sequences, with only a positive charge, penalizes the TFA salting. To further confirm the presence of β-sheet structures, two colorimetric tests, CR and ThT assays, were performed. CR is an azoic dye typically used as a probe to support the occurrence of amyloid-like fibrils.^41,42^ This assay is qualitatively considered positive when a CR solution, incubated with the peptide one, visibly changes its color (inset in Figure 4).

This spectral modification is also associated with the redshift of the absorbance peak (from 490 to ∼540 nm). As reported in Figure 4a, all the peptide solutions were found positive to the CR assay, thus again supporting the presence of β-sheets in the self-assembled nanostructures. The β-sheet amyloid-like structures were also evidenced by the ThT assay performed at the solid state.^43^ In detail, a few microliters of each sample solution was drop-casted on a slide glass. The dried film was then stained with a 50 μmol/L ThT solution. The resulting films, imaged by fluorescence microscopy, show an intense emission in the green spectral region (Figure 4b).

### Structural Characterization at the Solid State

#### SEM

**Figure 5:**
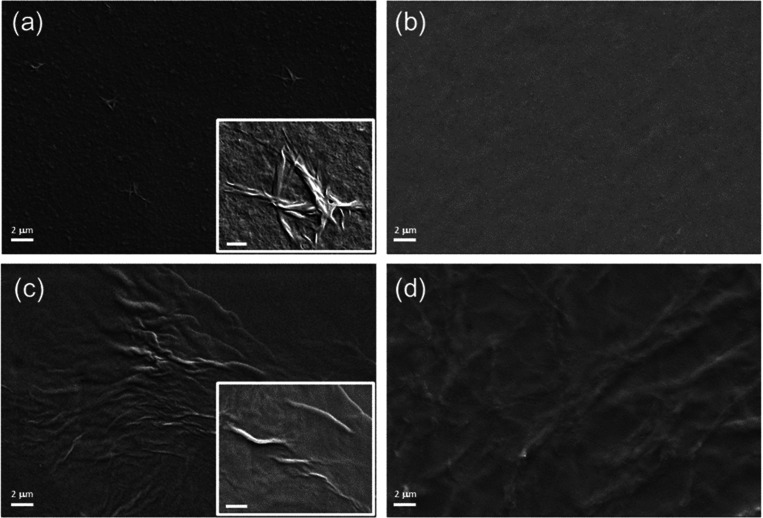
Selected SEM microphotos: (a) (WY)3, (b) PEG8-(WY)3, (c) (W-Dopa)3, and (d) PEG8-(W-Dopa)3. Scale bar: 2 μm. For the inset of parts (a) and (c), the scale bar is for 200 nm.

The morphology of the peptide aggregates was studied by the SEM technique. Representative microphotos, collected in Figure 5, were acquired on samples prepared by drop-casting water peptide solutions (at their maximum solubility) at a concentration higher than CACs. These conditions allow the visualization of the peptides in their self-assembled architectures, even if all of the samples can differ greatly in their relative concentration. To avoid possible interference of the aluminum stub surface with peptide aggregates, we drop-casted their aqueous solutions on inert glass slides. From the examination of Figure 5, a preferential formation of films for all the W-containing sequences can be observed. Moreover, some of them are characterized by the presence of clusters and evident supramolecular architectures. Specifically, irregular conglomerates formed by short elongated twisted fibers (measuring 200 nm in thickness and a few micrometers in length) are clearly visible in the film of (WY)3 (Figure 5a and its inset). These structures are not detectable in the PEGylated version of (WY)3 (Figure 5b). It may be supposed that this different behavior can be attributed to the capability of the PEG to alter the wrapping of the β-sheet,^44,45^ discoursing defects and forming more homogeneous surfaces, reducing the tendency of fiber formation in these conditions.

More elongated interconnected twisted fibers were found in the (W-Dopa)3 sample (Figure 5c and its inset). A quite similar network is also present in PEG8-(W-Dopa)3, which is incorporated in the film layer (Figure 5d). The found morphologies are in good agreement with the expected ones for fibrillary architectures, even if the formation of films prevails under these experimental conditions.

#### Wide-Angle X-ray Scattering (WAXS/GIWAXS)

**Figure 6:**
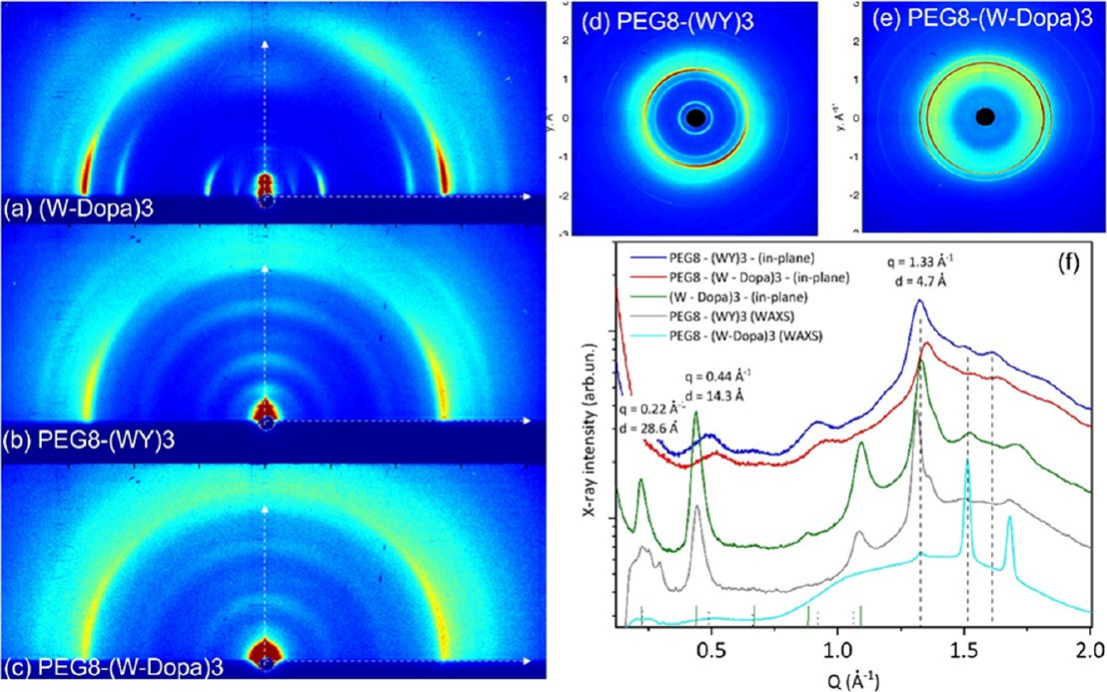
Left column: (a) (W-Dopa)3, (b) PEG8-(WY)3, and (c) PEG8-(W-Dopa)3 2D GIWAXS patterns. The out-of-plane and in-plane directions are marked with vertical and horizontal white arrows, respectively. Right columns: (d) PEG8-(WY)3 and (e) PEG8-(W-Dopa)3 2D WAXS patterns measured on solid fibers. (f) 1D linear cuts representing the scattered X-ray intensity along the in-plane and out-of-plane directions for GIWAXS and WAXS. The peak positions are reported in Tables 3 and 4.

Further structural insight into the nanofibers was obtained by wide-angle X-ray scattering in transmission (WAXS) and grazing incidence (GIWAXS) geometry, respectively. The reflection geometry and the preferred orientation of fibers induced by the substrate allow us to recognize the scattered X-ray intensity distribution along different directions in GIWAXS data (Figure 6, left column), i.e., perpendicular (out-of-plane) and parallel (in-plane) to the sample plane. Such directions correspond to different directions across the fiber structure, as seen in particular in the case of (W-Dopa)3 (Figure 6a), which features the highest preferred orientation. Most of the scattered intensities are indeed distributed along the in-plane direction and concentrated in diffraction peaks at *Q* = 0.22, 0.44, 1.09, and 1.33 Å^–1^, corresponding to the *d*-spacings of 28.6, 14.3, 5.7, and 4.7 Å, respectively; minor contributions are visible at *Q* = 0.67, 0.88, 1.52, 1.63, and 1.71 Å^–1^, corresponding to the *d*-spacings of 9.4, 7.2, 4.1, 3.8, and 3.7 Å, respectively. It can be recognized that all peaks up to *q* = 1.1 Å^–1^ are equally spaced by Δ*Q* = 0.22 Å^–1^ and can be therefore related to the same structural periodicity *d* = 2π/Δ*Q* = 2.86 nm along the fiber axis. The diffraction peaks appearing at larger scattering angles are related to the molecular structure (in particular to the interatomic distances of 4.7, 4.1, 3.8, and 3.7 Å).

**Table 3:** WAXS Reflections (*Q* [Å^–1^]) and *d*-Spacing (*d* [Å])

	reflections	
compound	*Q* [Å^–1^] ± 0.02	*d* [Å] ± 0.5
PEG8-(WY)	0.22	**28.6**
	0.44	14.3
	1.1	5.7
	1.33	4.7
	1.52	4.1
	1.7	3.7
	0.22	**28.6**
	1.33	4.7
PEG8-(W-Dopa)3	1.52	4.1
	1.7	3.7

**Table 4:** GIWAXS Reflections (*Q* [Å^–1^]) and *d*-Spacing (*d* [Å]) along the In-Plane and Out-of-Plane Directions^a^

	in-plane reflections		out-of-plane reflections	
compound	*Q* [Å^–1^] ± 0.02	*d* [Å] ± 0.5	*Q* [Å^–1^] ± 0.02	*d* [Å] ± 0.5
PEG8-(WY)3	0.25	25.0	0.25	25.0
	(0.44)	(14.3)	(0.44)	(14.3)
	0.49	13.0	0.49	13.0
	0.67	9.3	0.67	9.3
	0.91	6.9	0.91	6.9
	1.05	6.0	1.05	6.0
	1.19	5.3	**1.23**	**5.1**
	**1.33**	**4.7**	1.33	4.7
	1.43	4.4	1.43	4.4
	1.51	4.2		
	**1.61**	**3.9**		
	1.82	3.4		
PEG8-(W-Dopa)3	0.26	24.0	0.26	24.0
	(0.44)	(14.3)	(0.44)	(14.3)
	0.49	13.0	0.49	13.0
	0.67	9.3	0.67	9.35
	0.92	6.8	0.91	6.89
	1.07	5.9	1.07	5.9
	1.2	5.2	1.2	5.2
	**1.33**	**4.7**	(1.33)	(4.7)
	1.52	4.1		
	1.63	3.8		
	1.83	3.4		
(W-Dopa)3	**0.22**	**28.6**	(0.22)	(28.6)
			0.36	17.2
	**0.44**	**14.3**		
			0.50	12.5
			(0.61)	(10.3)
	(0.67)	(9.4)		
	0.88	7.2		
			**0.95**	**6.6**
	1.09	5.7		
	**1.33**	**4.7**	1.33	4.7
	1.52	4.1		
	1.63	3.8		
	1.71	3.7		

a The most and least intense reflections are reported in bold and in brackets, respectively.

They are all shared with the other two samples, PEG8-(W-Dopa)3 and PEG8-(WY)3 (as marked by the dashed black bars in Figure 6f, except for the last value 3.7 Å). A broad spot around *Q* = 0.94 Å^–1^ in the orthogonal (i.e., out-of-plane) direction reveals the *d*-spacing (6.7 Å periodicity) between the residues of the β-chains in (W-Dopa)3 (Figure 6a and Figure S8d). Moreover, both the PEGylated peptides and PEG8-(WY)3 (Figure 6b) and PEG8-(W-Dopa)3 (Figure 6c) samples also show a preferred orientation, but with a lower degree compared to the unPEGylated one (W-Dopa)3, as it can be recognized by the main diffraction ring becoming continuous along the azimuth in PEG8-(W-Dopa)3 (Figure 6c).

Also, when comparing 1D GIWAXS profiles extracted along the in-plane and out-of-plane directions (Figure 6f and Figure S8b–d), very similar features result up to *Q* = 1.1 Å^–1^ in the PEG8-(W-Dopa)3 and PEG8-(WY)3 samples, showing broad peaks due to diffraction rings more extended along the azimuth and related to slightly different periodicities (2.50 and possibly 2.86 nm). On the other hand, all samples basically share the same diffraction peaks at *Q* larger than 1.2 Å^–1^, hence related to the shortest interatomic distances. The common 28.6 Å periodicity is confirmed by WAXS measurements on free-standing fibers (Figure 6d,e). The diffraction peak at *Q* = 0.44 Å^–1^ (second-order diffraction for the 28.6 Å periodicity) in Figure 6 is indeed found with a significant intensity both in the GIWAXS pattern (where the first-order diffraction at *Q* = 0.22 Å^–1^ is also clearly detected) of deposited (W-Dopa)3 and in the WAXS pattern of the free-standing PEG8-(WY)3 fibers. These samples also show very similar diffraction profiles at larger *Q* values (i.e., similar molecular structure). On the contrary, the free-standing PEG8-(W-Dopa)3 fibers show much broader and low-intensity peaks at lower *Q* values (i.e., a much lower nanoscale order), similar to the deposited PEG8-(WY)3 and PEG8-(W-Dopa)3 fibers, but much sharper and intense peaks at large *Q* values. Based on the comparison between WAXS and GIWAXS patterns, it can be concluded that (W-Dopa)3 consists of long ordered supported fibers that preferentially lay parallel to the substrate plane; on the other hand, PEG8-(WY)3 only forms long ordered free-standing fibers, but when deposited on a substrate, they are expected to form smaller crystalline domains at the nanoscale, which are less constrained in the substrate plane and therefore orient rather randomly; a similar argument holds for PEG8-(W-Dopa)3, although some differences at the atomic scale are also visible by comparing WAXS and GIWAXS measurements, affecting relative peak intensities and their fwhm (full width at half-maximum). Finally, the 2D GIWAXS pattern of the (WY)3 peptide (Figure S9a) and the corresponding 1D linear cut (Figure S9b) are characterized by two broad rings without any preferred orientations, although a well-defined periodicity of 13 Å is detected (*Q* = 0.48 Å^–1^). Since there is generally good agreement between the most relevant peaks in the scattergrams of peptides endowed or not with PEG moieties, we can exclude a contribution to scattering from PEG crystallization. It is worth noting that PEG is a highly flexible chemical entity that has been never found to be structured in the crystal structures of PEG-derivatized globular proteins.^46^

#### Hydrogel Formation Tests and Rheological Characterization

**Figure 7:**
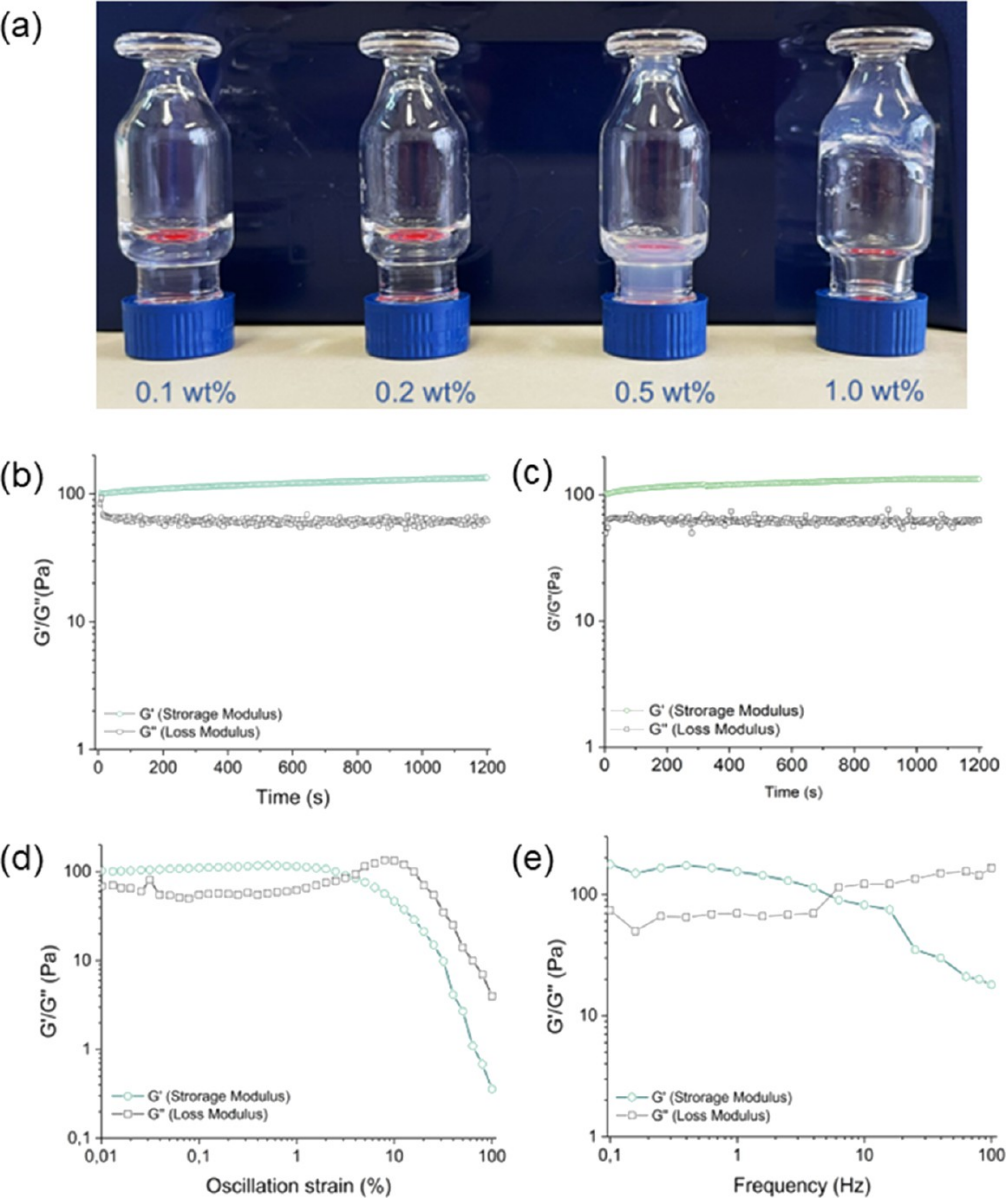
Hydrogel formulation and rheological analysis: (a) inverted test tube for the (WY)3 peptide at different concentrations. Rheological analysis of (WY)3: (b, c) time sweeps in duplicate showing the hydrogel storage modulus (*G*′) and loss modulus (*G*″), (d) dynamic strain sweep oscillatory test performed at a 1 Hz frequency, and (e) dynamic frequency sweep oscillatory test at a 0.1% strain.

The ability of peptides to form hydrogels according to the solvent-switch procedure, previously used for other aromatic peptides,^27,32^ was investigated. The solvent-switch procedure consists of the dilution in water (antisolvent phase) of the peptide previously dissolved at a very high concentration (100 mg/mL) into an organic solvent, generally dimethyl sulfoxide (DMSO). At a final concentration of 1.0 wt % (10 mg/mL), only the (WY)3 peptide generates a matrix with self-supporting features. No syneresis phenomena were detected, indicating that water is totally confined to the supramolecular architecture. The critical gelation concentration (CGC), identified using an inverted test tube (see Figure 7a), was in the 0.5 < CGC < 1.0 wt % range. Additionally, the supramolecular system generated by (WY)3 exhibits swelling properties (*q*% = 22%), further suggesting the capability of the peptide to form a hydrogel. To analytically confirm the gel state of the sample, a rheological analysis was executed. Twenty minute time-sweep oscillatory tests (frequency ν = 1.0 Hz and strain ω = 0.1%, Figure 7b,c in duplicate) were performed using a rotational plate geometry rheometer and plotting results in terms of *G*′ (storage modulus) and *G*″ (loss modulus). Stability parameters were identified by performing preliminary dynamic oscillation strain sweep (ν = 1.0 Hz) and dynamic frequency sweep (ω = 0.1%) tests (Figure 7c,d). The LVE region (linear viscoelastic range) was found in the 0.01–2.6% range with a yield point of 4.3%. From the inspection of modulus values (*G*′ = 134 Pa; *G*″ = 62 Pa), the soft mechanical nature of the matrix is noticeable, as also confirmed by the frequency break point of 5.5 Hz and a tan δ (*G*′/*G*″) = 2.16. The soft nature of the system can also explain the difference (less than 1 order of magnitude) between storage and loss modulus values.

The mechanical response of (WY)3 is similar to the previously studied PEG8-(FY)3 sequence (*G*′ = 100 Pa; *G*″ = 28 Pa).^26^ Compared to other all-aromatic peptide-based hydrogels [(Nal-Y)3, 335 Pa; (F-Dopa)3, 60 Pa; and (Nal-Dopa)3, 730 Pa] containing Nal and/or Dopa residues in their primary sequence,^27^ gel formation is possible only for the unPEGylated version of the primary sequence. The rheological response of (WY)3 compared to (Nal-Y)3, with a 3-fold reduction of *G*′, may suggest reduced entanglements of the constitutive fibers for the W-containing sequences.

#### MD Simulations

**Figure 8:**
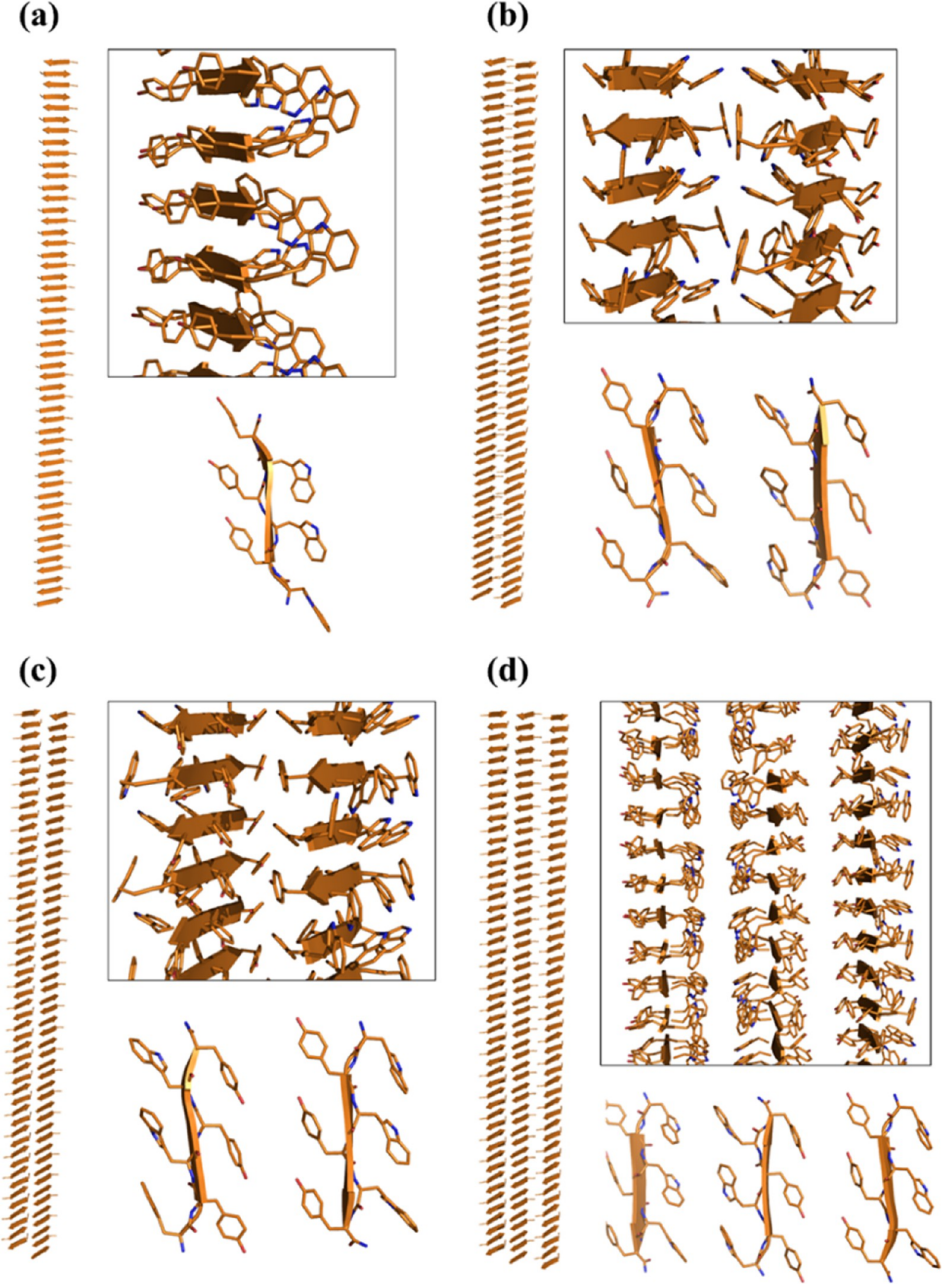
Three-dimensional representations of the starting flat models of (WY)3 aggregates used in the MD studies: (a) WY_ST50_SH1, (b) WY_ST50_SH2_WW, (c) WY_ST50_ SH2_YY, and (d) WY_ST50_SH3.

To gain atomic-level structural data on the peptide moiety of the assemblies formed by (WY)3, we carried out molecular modeling and MD studies. This model was chosen as it contains only standard amino acid residues, for which the parameters of MD force fields have been extensively validated, and no other chemical modification. As detailed in the Experimental Section, different models composed of one, two, or three β-sheets, each containing 50 β-strands, were generated. Representations of the starting models are shown in Figure 8. Initial MD investigations were performed on a single β-sheet made of 50 β-strands (WY_ST50_SH1; Figure 8a).

#### Stability and Rigidity of the Cross-β Assemblies

The evaluation of the geometrical parameters that are commonly used to assess the structural stability of trajectory structures (RSMD deviations from the starting model and radius of gyration, *R*_g_) indicates that the single β-sheet system undergoes major structural rearrangements in the simulation time scale (Figure S10a,b). Although the flat starting model is not preserved, the inspection of Figure S10c indicates that the secondary structure of the system is rather locally conserved. Altogether, these findings show that (WY)3 is endowed with a propensity to form β-structures, although the high RMSD values (up to 25 Å) and the strong decrease of *R*_g_ (about 10 Å) indicate that the overall structure of the single sheet is unstable.

Then, we evaluated the possibility that (WY)3 could form assemblies through the tight lateral association of either Trp or Tyr side chains. To this aim, we generated two distinct double-sheet models (WY_ST50_SH2_WW and WY_ST50_SH2_YY) illustrated in Figure 8b,c that were used as starting structures in MD simulations. The inspection of the trajectory frames of the resulting simulations clearly indicates that, for both systems, after an initial structural transition occurring in the very first part of the simulation (within 20 ns), the models reach rather stable structural states (Figures S11a,b and S12a,b). The analysis of the individual trajectory frames demonstrates that the initial structural transition corresponds to the twisting of the originally flat models whose secondary structure is, however, rather well preserved (Figures S11c and S12c). The evaluation of the flexibility through the calculation of the RMSF values in the 50–200 ns portion of the trajectories indicates that (WY)3 residues, both those located at the intersheet interface and those exposed to the solvent, are endowed with limited mobility (Figures S11d and S12d).

**Figure 9:**
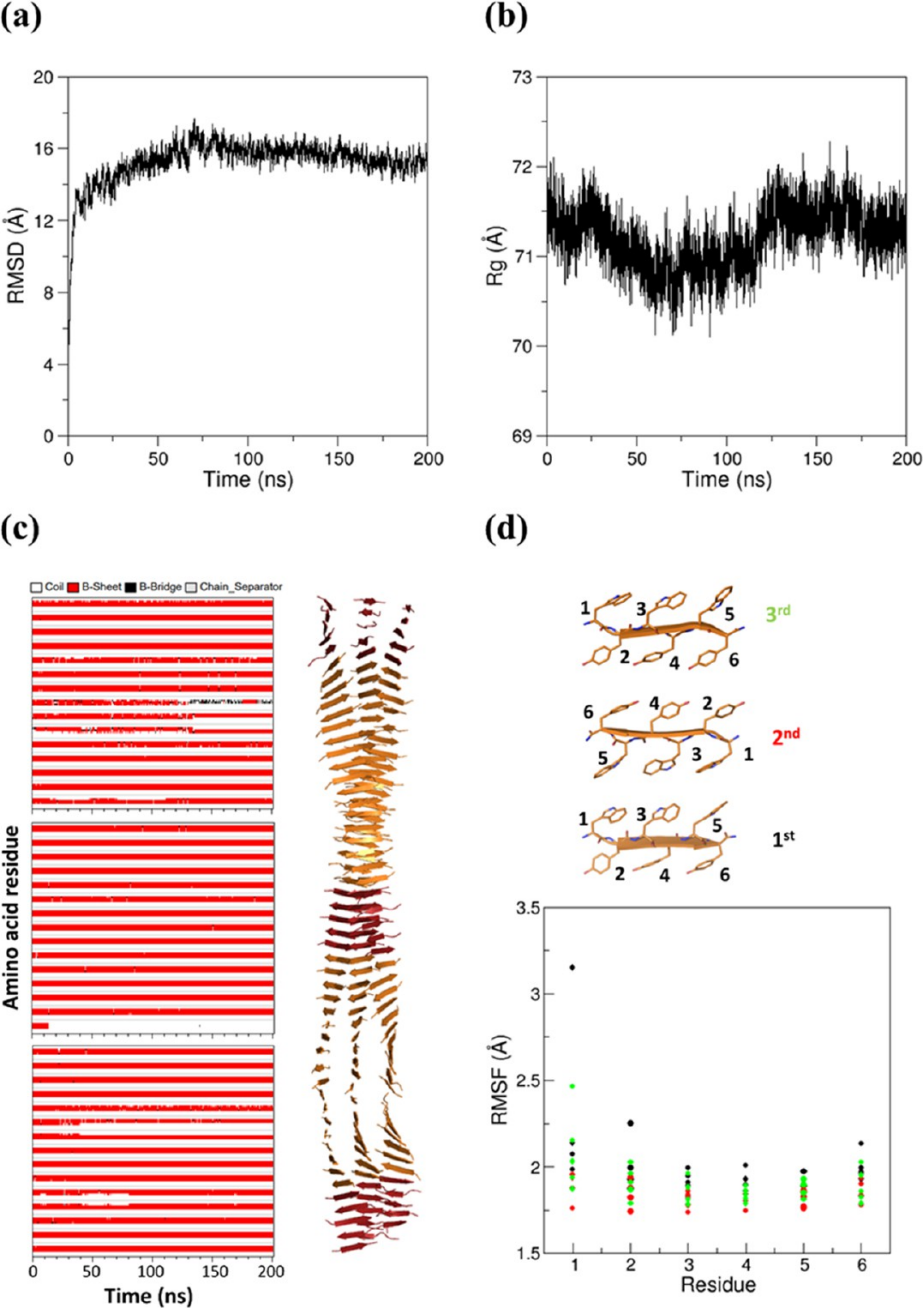
MD parameters evaluated in the simulation of WY_ST50_SH3: (a) RMSD values of trajectory structures against the starting flat model computed on the C^α^ atoms, (b) gyration radius *R*_g_, (c) secondary structure time evolution, and (d) RMSF values computed on the C^α^ atoms of the 15 (five per sheet) central β-strands in the equilibrated region of the trajectory (50–200 ns). For the sake of clarity, the secondary structure is reported only for the residues belonging to the terminal ends and to the central region. The chain separator (light gray) shows the interruption in the polypeptide chain, thus allowing us to discern the different strands composing the β-sheets. A cartoon representation of the twisted average structure is also reported in panel (c). A stick representation of a triplet of facing β-strands is reported in panel (d).

The ability of both Trp and Tyr residues of (WY)3 to tightly associate to form stable interfaces, as shown in the previous paragraph, allowed us to generate a three-sheet model characterized by two distinct interfaces: one made of Trp and the other one made of Tyr residues (WY_ST50_SH3; Figure 8d). MD simulations conducted on this more complex system clearly indicate that this assembly is quite stable, as shown by the time evolution of several structural parameters (RMSD, *R*_g_, and secondary structure; Figure 9a–c). The model is also endowed with significant rigidity, as indicated by the low RMSF values of both Trp and Tyr residues (Figure 9d). As observed for the double-sheet models, the initial flat structure undergoes a twisting while its secondary structure is preserved (Figure 9c).

#### Interatomic Distances of the Cross-β Assemblies: Quantification and Evolution

**Figure 10:**
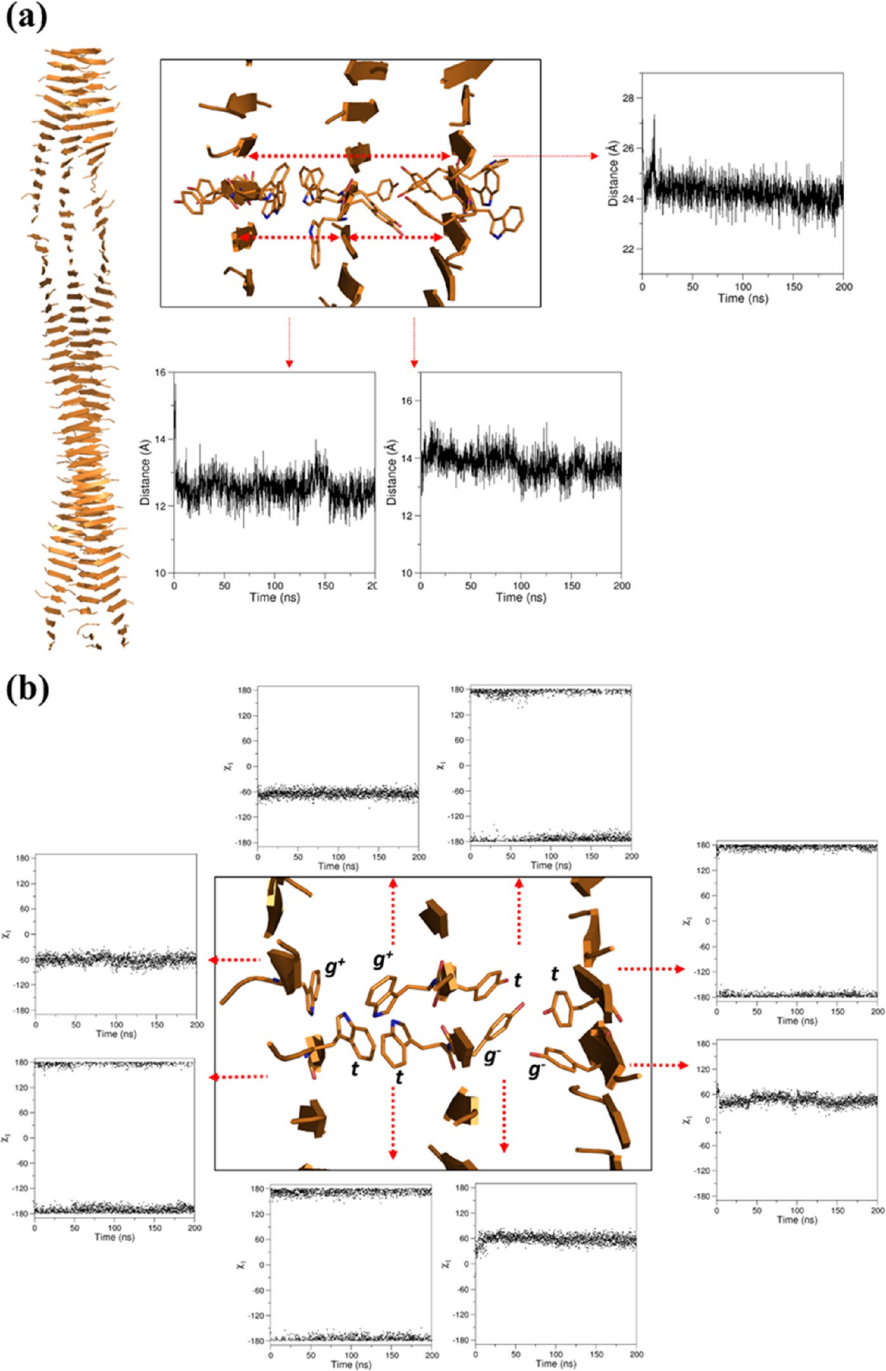
Representative examples of the time evolution of (a) the distances between two selected C^α^ atoms of the β-sheets and (b) the χ1 dihedral angle of Trp/Tyr side chains in the simulation of WY_ST50_SH3.

**Figure 11:**
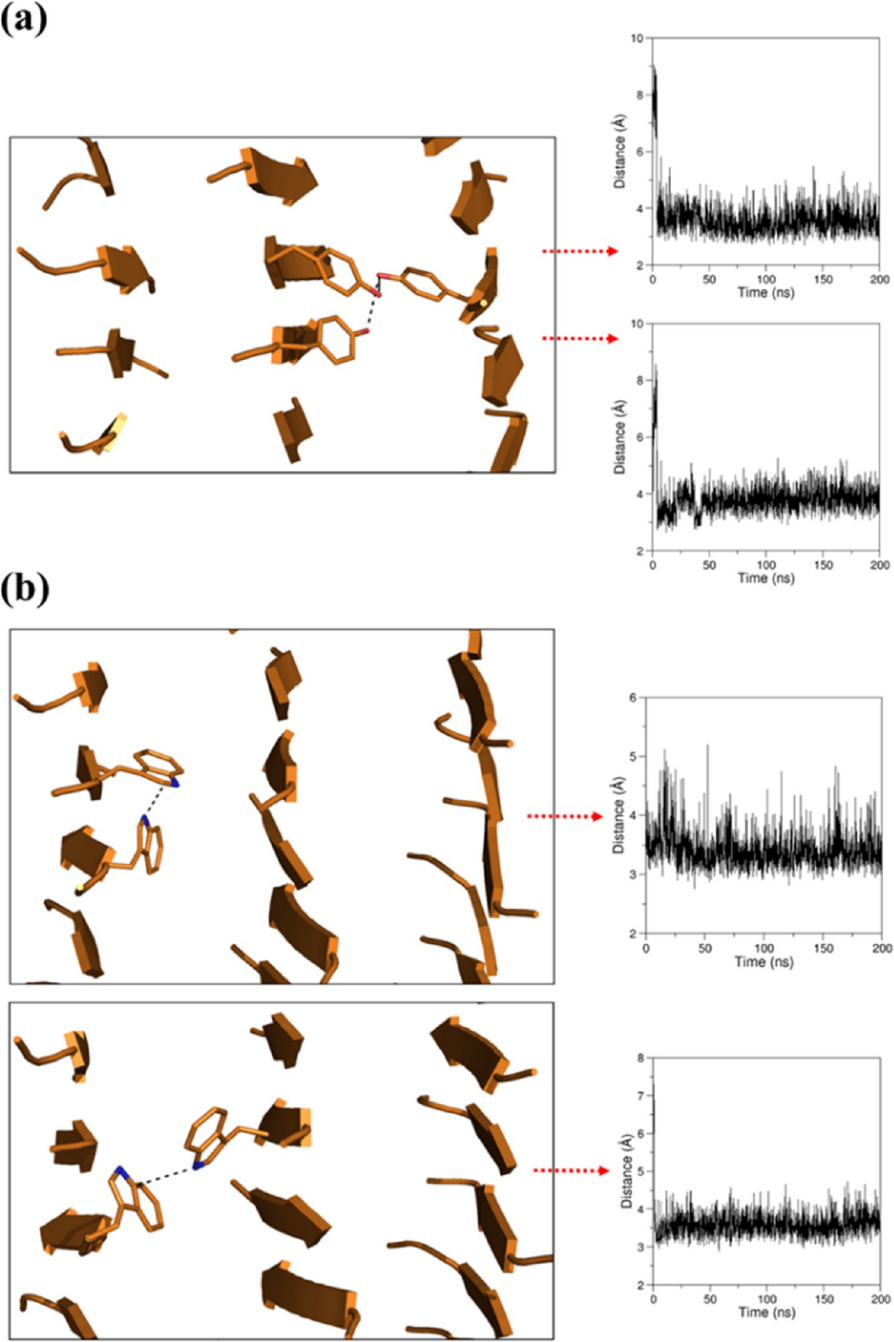
Representative examples of (a) intersheet H-bonding interactions at the Tyr–Tyr interface and (b) intra- and intersheet N−π interaction at the Trp–Trp interface of WY_ST50_SH3. The time evolution of the distances between the OH atoms (for Tyr) and NE1-CE2 atoms (for Trp) in the MD simulation is reported.

The cross-β structure is stabilized by several interatomic interactions that are well preserved throughout the MD simulations. In addition to the network of backbone hydrogen bonds that stabilizes the β-structure and generates the periodicity at 4.7 Å, which is parallel to the growth axis of the assembly and is the defining feature of the cross-β motif, the supramolecular organization of (WY)3 also relies on intersheet distances with a periodicity of approximately 13–14 Å that is detected at both Trp–Trp and Tyr–Tyr interfaces (Figure 10a). The combination of these two interfaces leads to a structure characterized by a periodicity of approximately 25 Å (Figure 10a). The structural analysis of both interfaces revealed that this system is characterized by alternation of *trans* and *gauche* rotameric states of the side chains within each strand (Figure 10b). This recurrent structural organization leads to the formation of stabilizing interactions at both the intra- and intersheet sides. In particular, the Tyr–Tyr interface is stabilized by H-bonds formed by the OH groups present in the Tyr side chain (Figure 11a), whereas N−π interactions involving the N^ε1^ atom present in the side chain of one Trp and the aromatic ring of an adjacent Trp residue led to the stabilization of the Trp–Trp interface (Figure 11b).

**Figure 12:**
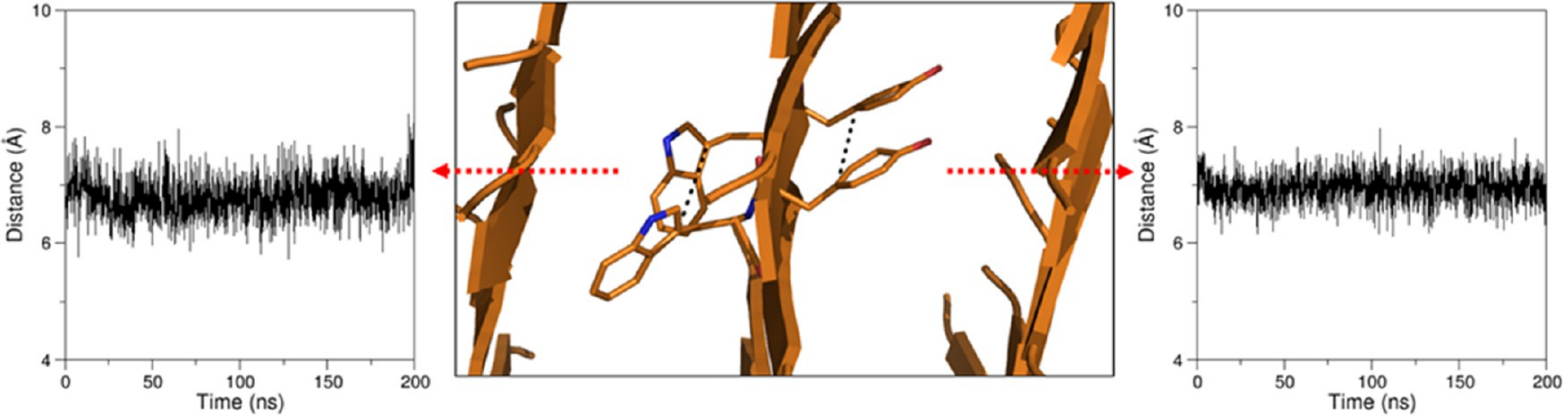
Representative examples of the time evolution of the distances between the C^γ^ atoms of the Trp (on the left) or Tyr (on the right) side chains of the central β-sheet in the simulation of WY_ST50_SH3.

The rigidity of the bulky side chains of these aromatic residues, which is produced by these strong and well-defined interactions, also generates a periodicity that is slightly below 7 Å along the peptide chain. This is likely due to the characteristic alternating orientation of the residue side chains in the β-structure (Figure 12).

#### Structural Interpretation of the WAXS/GIWAXS Data

**Figure 13:**
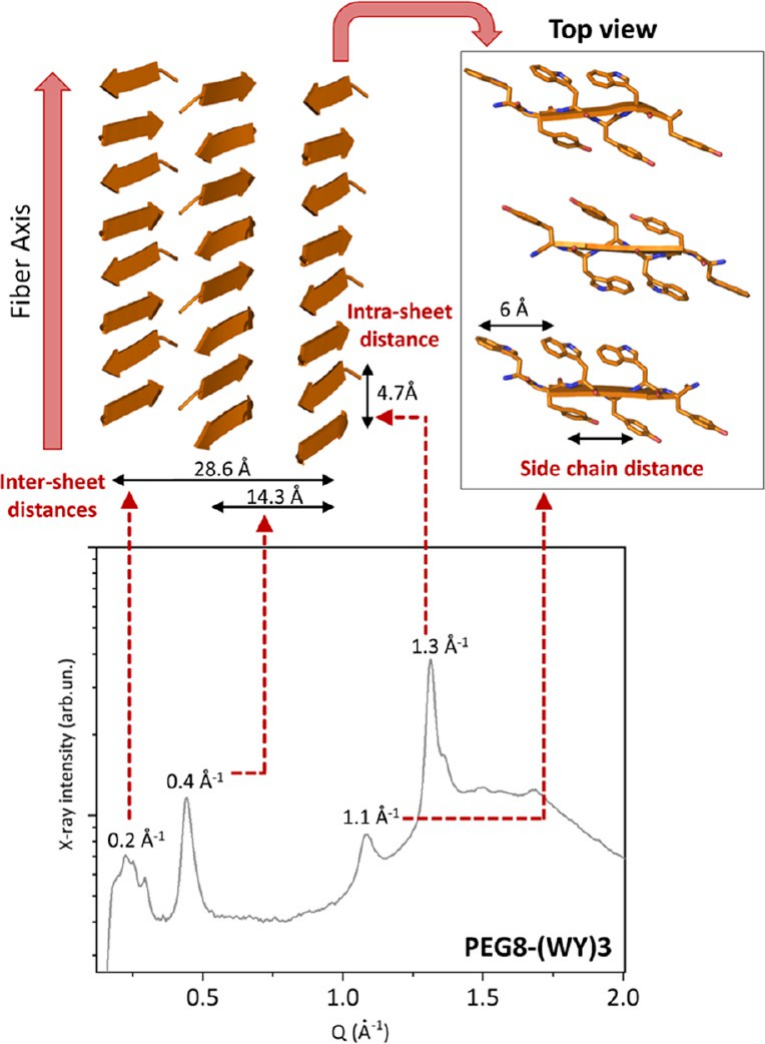
Structural interpretation of the WAXS/GIWAXS data. The correspondence between the peaks detected in the WAXS pattern of PEG8-(WY)3 and the recurrent distances in the three-dimensional model is shown.

The main geometrical periodicity of the three-dimensional models that emerged from the MD simulations is in good agreement with those retrieved from the experimental characterization of the (WY)3 samples. Indeed, the WAXS pattern of this compound is characterized by the presence of two peaks centered at *d*-spacings of 4.7 and 14.3 Å, which correspond in the atomic model of the assembly to the β-stands and the intersheet distances, respectively (Figure 13). These features, with limited variations, also emerge from the WAXS and GIWAXS analyses of the other compounds. Assuming that the structured spine of (W-Dopa)3, which presents the highest preferred orientation on the substrate, resembles that of (WY)3, an atomic-level interpretation of the GIWAXS data for the assemblies formed by this compound may be attempted.

Indeed, the periodicities observed along the in-plane direction correspond to the elongation axis of assemblies (4.7 Å) and to the intersheet distances (∼14 and 28 Å). On the other hand, the only significant feature observed out-of-plane is detected at 6–7 Å, which likely corresponds to the direction of the peptide chains with the observed periodicity generated by the alternating side chains of the aromatic residues (Figure 13). In this scenario, the network of hydrogen bonds and the intersheet interfaces lies on the substrate with the covalent peptide chain running perpendicularly to it.

## Conclusions

Peptides endowed with a completely aromatic sequence such as (FY)3 and PEG8-(FY)3 have been recently identified as suitable building blocks for the formulation of soft hydrogels.^26^ MD studies demonstrated that the interconnected fibrillary networks are originated by the capability of these peptides to opportunely arrange by forming two interfaces: a wet and a dry one. In the attempt to identify novel materials with enhanced and unexpected properties,^47^ we punctually replaced the original, natural amino acids with the non-natural ones like Nal and Dopa.^27^ The effect of these modifications and of the PEG moiety at the N-terminus of the peptides were evaluated.^27,48^

Here, we replaced Phe residues with Trp residues, obtaining novel peptide sequences [(WY)3 and (W-Dopa)3]. The structural characterization of these peptides, performed both in solution and in the solid state, allowed us to conclude that all the peptides are able to self-assemble into fibrillary nanostructures, in which the peptide sequences are organized into β-sheet structures. Among them, only (WY)3 keeps the capability to generate soft hydrogels with a *G*′ value of 134 Pa similar to the other completely aromatic hexapeptides previously studied under the same conditions [PEG8-(FY)3, (Nal-Y)3, (F-Dopa)3, and (Nal-Dopa)3]. The MD studies carried out on the (WY)3 peptide provide interesting insights into the atomic structure of the cross-β that constitutes the basic motif of the assemblies formed by this peptide. Interestingly, these analyses clearly show that both the Tyr–Tyr and Trp–Trp interfaces are characterized by precise juxtaposition of the side chains of these residues, as demonstrated by the unique rotameric states they assume. This overall rigidity is generated by strong interactions that characterize both the Tyr–Tyr (hydrogen bonds) and the Trp–Trp (N−π interaction) interfaces. Both Trp and Dopa represent also possible sites for postaggregation modification, thus including metal chelation, redox-responsiveness, and chemical cross-linking points. In this scenario, the sequences characterized here can be exploited as tunable tools for developing soft hydrogels aimed at expanding the applications of peptide materials.

## Supporting Information Available

(S1–S4) Chemical formula, chromatographic RP-HPLC profile, and ESI mass spectrum of W-containing peptides. (S5) UV–vis spectra of peptides. (S6) Fluorescence emission spectra of the aromatic peptides at different concentrations. (S7) First derivative functions in the 2000–1000 cm^–1^ IR range. (S8) GIWAXS profiles. (S9) 2D GIWAXS patterns of the (WY)3 peptide and of the corresponding 1D linear cut representing the scattered X-ray intensity. (S10) MD parameters evaluated in the simulation of WY_ST50_SH1: RMSD values of trajectory structures against the starting flat model computed on the C^α^ atoms, gyration radius, and secondary structure time evolution. (S11) MD parameters evaluated in the simulation of WY_ST50_SH2_WW: RMSD values of trajectory structures against the starting flat model computed on the C^α^ atoms, gyration radius *R*_g_, secondary structure time evolution, and RMSF values computed on the C^α^ atoms of the ten (five per sheet) central β-strands in the equilibrated region of the trajectory (50–200 ns). (S12) MD parameters evaluated in the simulation of WY_ST50_SH2_WW: RMSD values of trajectory structures against the starting flat model computed on the C^α^ atoms, gyration radius *R*_g_, secondary structure time evolution, and RMSF values computed on the C^α^ atoms of the ten (five per sheet) central β-strands in the equilibrated region of the trajectory (50–200 ns). (Table S1) MD simulation parameters. (Table S2) Deconvolution percentage functions of secondary structures in the amide I region (PDF)

## References

[ref1] Jhaveri A. M.; Torchilin V. P. Multifunctional polymeric micelles for delivery of drugs and siRNA. Front. Pharmacol. 2014, 5, 1–26. 10.3389/fphar.2014.00077. 24795633 PMC4007015

[ref2] Miranda M. S.; Almeida A. F.; Gomes M. E.; Rodrigues M. T. Magnetic micellar nanovehicles: prospects of multifunctional hybrid systems for precision theranostics. Int. J. Mol. Sci. 2022, 23 (19), 11793 10.3390/ijms231911793. 36233094 PMC9569989

[ref3] Pellegrino P.; Bramanti A. P.; Farella I.; Cascione M.; De Matteis V.; Della Torre A.; Quaranta F.; Rinaldi R. Pulse-atomic force lithography: a powerful nanofabrication technique to fabricate constant and varying-depth nanostructures. Nanomaterials 2022, 12 (6), 991 10.3390/nano12060991. 35335805 PMC8953364

[ref4] Charumathy A.; Ubaidulla U.; Sinha P.; Rathnam G. Recent update on liposome-based drug delivery system. Int. J. Curr. Pharm. Res. 2022, 14 (3), 22–27. 10.22159/ijcpr.2022v14i3.1991.

[ref5] Sideri I. K.; Tagmatarchis N. Noble-metal-free doped carbon nanomaterial electrocatalysts *Chem*. Eur. J. 2020, 26 (67), 15397–15415. 10.1002/chem.202003613. 32931046

[ref6] Parisi E.; Adorinni S.; Garcia A. M.; Kralj S.; De Zorzi R.; Marchesan S. Self-assembling tripeptide forming water-bound channels and hydrogels. J. Peptide Sci. 2023, e3524 10.1002/psc.3524. 37226306

[ref7] Evers M. J. W.; van de Wakker S. I.; de Groot E. M.; de Jong O. G.; Gitz-François J. J. J.; Seinen C. S.; Sluijter J. P. G.; Schiffelers R. M.; Vader P. Functional siRNA delivery by extracellular vesicle–liposome hybrid nanoparticles. Adv. Healthcare Mater. 2022, 11, 2101202 10.1002/adhm.202101202. PMC11468224 34382360

[ref8] Arkan E.; Azandaryani A. H.; Moradipour P.; Behbood L. Biomacromolecular based fibers in nanomedicine: a combination of drug delivery and tissue engineering. Curr. Pharm. Biotechnol. 2018, 18 (11), 909–924. 10.2174/1389201019666180112144759. 29332574

[ref9] Lu F.; Wang M.; Li N.; Tang B. Polyoxometalate-based nanomaterials toward efficient cancer diagnosis and therapy. Chem. - Eur. J. 2021, 27 (21), 6422–6434. 10.1002/chem.202004500. 33314442

[ref10] Lock L. L.; Li Y.; Mao X.; Chen H.; Staedtke V.; Bai R.; Ma W.; Lin R.; Li Y.; Liu G.; Cui H. One-component supramolecular filament hydrogels as theranostic label-free Magnetic Resonance Imaging agents. ACS Nano 2017, 11 (1), 797–805. 10.1021/acsnano.6b07196. 28075559 PMC5773287

[ref11] Binaymotlagh R.; Chronopoulou L.; Haghighi H. F.; Fratoddi I.; Palocci C. Peptide-based hydrogels: new materials for biosensing and biomedical applications. Materials 2022, 15 (17), 5871 10.3390/ma15175871. 36079250 PMC9456777

[ref12] Elsawy M. A.; Wychowaniec J. K.; Castillo Diaz L. A.; Smith A. M.; Miller A. F.; Saiani A. Controlling doxorubicin release from a peptide hydrogel through fine-tuning of drug–peptide fiber interactions. Biomacromolecules 2022, 23 (6), 2624–2634. 10.1021/acs.biomac.2c00356. 35543610 PMC9198986

[ref13] Nummelin S.; Liljeström V.; Saarikoski E.; Ropponen J.; Nykänen A.; Linko V.; Seppälä J.; Hirvonen J.; Ikkala O.; Bimbo L. M.; Kostiainen M. A. Self-assembly of amphiphilic janus dendrimers into mechanically robust supramolecular hydrogels for sustained drug release. Chem. - Eur. J. 2015, 21, 14433–14439. 10.1002/chem.201501812. 26134175

[ref14] Draper E. R.; Adams D. J. Low-molecular-weight gels: the state of the art. Chem. 2017, 3, 390–410. 10.1016/j.chempr.2017.07.012.

[ref15] Cimino R.; Gatto E.; De Zotti M.; Formaggio F.; Toniolo C.; Giannetti M.; Palleschi A.; Serpa C.; Venanzi M. Peptide-bridged bis-porphyrin compounds: a photophysical and molecular dynamics study. J. Photochem. Photobiol. 2023, 16, 100191 10.1016/j.jpap.2023.100191.

[ref16] Swanekamp R. J.; Welch J. J.; Nilsson B. L. Proteolytic stability of amphipathic peptide hydrogels composed of self-assembled pleated β-sheet or coassembled rippled β-sheet fibrils. Chem. Commun. 2014, 50, 10133–10136. 10.1039/C4CC04644G. 25050628

[ref17] Fleming S.; Ulijn R. V. Design of nanostructures based on aromatic peptide amphiphiles. Chem. Soc. Rev. 2014, 43 (23), 8150–8177. 10.1039/C4CS00247D. 25199102

[ref18] Hiew S. H.; Lu Y.; Han H.; Gonçalves R. A.; Alfarano S. R.; Mezzenga R.; Parikh A. N.; Mu Y.; Miserez A. Modulation of mechanical properties of short bioinspired peptide materials by single amino-acid mutations. J. Am. Chem. Soc. 2023, 145 (6), 3382–3393. 10.1021/jacs.2c09853. 36730942

[ref19] Reches M.; Gazit E. Casting metal nanowires within discrete self-assembled peptide nanotubes. Science 2003, 300 (5619), 625–627. 10.1126/science.1082387. 12714741

[ref20] Yan X.; Zhu P.; Li J. Self-assembly and application of diphenylalanine-based nanostructures. Chem. Soc. Rev. 2010, 39 (6), 1877–1890. 10.1039/b915765b. 20502791

[ref21] Marchesan S.; Vargiu A. V.; Styan K. E. The Phe-Phe motif for peptide self-assembly in nanomedicine. Molecules 2015, 20 (11), 19775–19788. 10.3390/molecules201119658. 26540034 PMC6332413

[ref22] Gnanasekaran K.; Korpanty J.; Berger O.; Hampu N.; Halperin-Sternfeld M.; Cohen-Gerassi D.; Adler-Abramovich L.; Gianneschi N. C. Dipeptide nanostructure assembly and dynamics via in situ liquid-phase electron microscopy. ACS Nano 2021, 15 (10), 16542–16551. 10.1021/acsnano.1c06130. 34623126 PMC9836046

[ref23] Schnaider L.; Brahmachari S.; Schmidt N. W.; Mensa B.; Shaham-Niv S.; Bychenko D.; Adler-Abramovich L.; Shimon L. J. W.; Kolusheva S.; DeGrado W. F.; Gazit E. Self-assembling dipeptide antibacterial nanostructures with membrane disrupting activity. Nat. Commun. 2017, 8 (1), 1–10. 10.1038/s41467-017-01447-x. 29118336 PMC5678095

[ref24] Diaferia C.; Sibillano T.; Balasco N.; Giannini C.; Roviello V.; Vitagliano L.; Morelli G.; Accardo A. Hierarchical analysis of self-assembled PEGylated hexaphenylalanine photoluminescent nanostructures. Chem. - Eur. J. 2016, 22 (46), 16586–16597. 10.1002/chem.201604107. 27706842

[ref25] Diaferia C.; Sibillano T.; Altamura D.; Roviello V.; Vitagliano L.; Giannini C.; Morelli G.; Accardo A. Structural characterization of PEGylated hexaphenylalanine nanostructures exhibiting green photoluminescence emission. Chem. - Eur. J. 2017, 23 (56), 14039–14048. 10.1002/chem.201703055. 28782843

[ref26] Diaferia C.; Balasco N.; Sibillano T.; Ghosh M.; Adler-Abramovich L.; Giannini C.; Vitagliano L.; Morelli G.; Accardo A. Amyloid-like fibrillary morphology originated by tyrosine-containing aromatic hexapeptides. Chem. - Eur. J. 2018, 24 (26), 6804–6817. 10.1002/chem.201800351. 29504716

[ref27] Diaferia C.; Netti F.; Ghosh M.; Sibillano T.; Giannini C.; Morelli G.; Adler-Abramovich L.; Accardo A. Bi-functional peptide-based 3D hydrogel-scaffolds. Soft Matter 2020, 16, 7006–7017. 10.1039/D0SM00825G. 32638818

[ref28] Birdi K. S.; Singh H.; Dalsager S.-U. Interaction of ionic micelles with the hydrophobic fluorescent probe 1-anilino-8-naphthalenesulfonate. J. Phys. Chem. 1979, 83, 2733–2737. 10.1021/j100484a010.

[ref29] Sunde M.; Serpell L. C.; Bartlam M.; Fraser P. E.; Pepys M. B.; Blake C. C. Common core structure of amyloid fibrils by synchrotron X-ray diffraction. J. Mol. Biol. 1997, 273, 729–739. 10.1006/jmbi.1997.1348. 9356260

[ref30] Altamura D.; Lassandro R.; Vittoria F. A.; De Caro L.; Siliqi D.; Ladisa M.; Giannini C. Rat-tail tendon fiber SAXS high-order diffraction peaks recovered by a superbright laboratory source and a novel restoration algorithm. J. Appl. Crystallogr. 2012, 45, 869–873. 10.1107/S0021889812025733.

[ref31] Sibillano T.; De Caro L.; Altamura D.; Siliqi D.; Ramella M.; Boccafoschi F.; Ciasca G.; Campi G.; Tirinato L.; Di Fabrizio E.; Giannini C. An optimized table-top small-angle X-ray scattering set-up for the nanoscale structural analysis of soft matter. Sci. Rep. 2014, 4, 6985 10.1038/srep06985. 25382272 PMC4225548

[ref32] Diaferia C.; Rosa E.; Balasco N.; Sibillano T.; Morelli G.; Giannini C.; Vitagliano L.; Accardo A. The introduction of a cysteine residue modulates the mechanical properties of aromatic-based solid aggregates and self-supporting hydrogels. Chem. - Eur. J. 2021, 27 (60), 14886–14898. 10.1002/chem.202102007. 34498321 PMC8596998

[ref33] Diaferia C.; Balasco N.; Sibillano T.; Giannini C.; Vitagliano L.; Morelli G.; Accardo A. Structural characterization of self-assembled tetra-tryptophan based nanostructures: variations on a common theme. ChemPhysChem 2018, 19 (13), 1635–1642. 10.1002/cphc.201800026. 29542851

[ref34] Colletier J. P.; Laganowsky A.; Landau M.; Zhao M.; Soriaga A. B.; Goldschmidt L.; Flot D.; Cascio D.; Sawaya M. R.; Eisenberg D. Molecular basis for amyloid-β polymorphism. Proc. Natl. Acad. Sci. U.S.A. 2011, 108 (41), 16938–16943. 10.1073/pnas.1112600108. 21949245 PMC3193189

[ref35] Van Der Spoel D.; Lindahl E.; Hess B.; Groenhof G.; Mark A. E.; Berendsen H. J. C. GROMACS: Fast, flexible, and free. J. Comput. Chem. 2005, 26, 1701–1718. 10.1002/jcc.20291. 16211538

[ref36] Humphrey W.; Dalke A.; Schulten K. VMD: Visual molecular dynamics. J. Mol. Graphics 1996, 14, 33–38. 10.1016/0263-7855(96)00018-5. 8744570

[ref37] Möller M.; Denicola A. Protein tryptophan accessibility studied by fluorescence quenching. Biochem. Mol. Bio. Ed. 2002, 30 (3), 175–178. 10.1002/bmb.2002.494030030035.

[ref38] Kong J.; Yu S. Fourier transform infrared spectroscopic analysis of protein secondary structures. Acta Biochim. Biophys. Sin. 2007, 39 (8), 549–559. 10.1111/j.1745-7270.2007.00320.x. 17687489

[ref39] Seo J.; Hoffmann W.; Warnke S.; Huang X.; Gewinner S.; Schöllkopf W.; Bowers M. T.; von Helden G.; Pagel K. An infrared spectroscopy approach to follow β-sheet formation in peptide amyloid assemblies. Nat. Chem. 2017, 9, 39–44. 10.1038/nchem.2615. 27995915

[ref40] Valenti L. E.; Paci M. B.; De Pauli C. P.; Giacomelli C. E. Infrared study of trifluoroacetic acid unpurified synthetic peptides in aqueous solution: trifluoroacetic acid removal and band assignment. Anal. Biochem. 2011, 410 (1), 118–123. 10.1016/j.ab.2010.11.006. 21078284

[ref41] Howie A. J.; Brewer D. B. Optical properties of amyloid stained by Congo Red: history and mechanisms. Micron 2009, 40 (3), 285–301. 10.1016/j.micron.2008.10.002. 19019688

[ref42] Klunk W. E.; Jacob R. F.; Mason R. P. Quantifying amyloid by congo red spectral shift assay. Methods Enzymol. 1999, 309, 285–305. 10.1016/S0076-6879(99)09021-7. 10507031

[ref43] Hudson S. A.; Ecroyd H.; Kee W. T.; Carver J. A. The thioflavin T fluorescence assay for amyloid fibril detection can be biased by the presence of exogenous compounds. FEBS J. 2009, 276, 5960–5972. 10.1111/j.1742-4658.2009.07307.x. 19754881

[ref44] Kar S.; Wu K.-W.; Hsu I.-J.; Lee C.-R.; Tai Y. Study of the nano-morphological versatility by self-assembly of a peptide mimetic molecule in response to physical and chemical stimuli. Chem. Commun. 2014, 50, 2638–2641. 10.1039/c3cc49769k. 24469335

[ref45] Cattani G.; Vogeley L.; Crowley P. B. Structure of a PEGylated protein reveals a highly porous double-helical assembly. Nat. Chem. 2015, 7, 823–828. 10.1038/nchem.2342. 26391082

[ref46] Diaferia C.; Rosa E.; Morelli G.; Accardo A. Fmoc-diphenylalanine hydrogels: optimization of preparation methods and structural insights. Pharmaceuticals 2022, 15, 1048 10.3390/ph15091048. 36145269 PMC9505424

[ref47] Balasco N.; Diaferia C.; Rosa E.; Monti A.; Ruvo M.; Doti N.; Vitagliano L. A comprehensive analysis of the intrinsic visible fluorescence emitted by peptide/protein amyloid-like assemblies. Int. J. Mol. Sci. 2023, 24 (9), 8372 10.3390/ijms24098372. 37176084 PMC10178990

[ref48] Diaferia C.; Mercurio F. A.; Giannini C.; Sibillano T.; Morelli G.; Leone M.; Accardo A. Self-assembly of PEGylated tetra-phenylalanine derivatives: structural insights from solution and solid state studies. Sci. Rep. 2016, 6, 26638 10.1038/srep26638. 27220817 PMC4879547

