# Supplementary material for: Self-Assembled Materials Based on Fully Aromatic Peptides: The Impact of Tryptophan, Tyrosine, and Dopa Residues
Source: Langmuir. 2024 Jan 4;40(2):1470–86. doi: 10.1021/acs.langmuir.3c03214 (PMC10795196; doi:10.1021/acs.langmuir.3c03214)
Supplement: Supplementary file 1 — la3c03214_si_001.pdf [file la3c03214_si_001.pdf]

Self-assembled materials based on fully aromatic peptides: the impact of tryptophan, tyrosine, and Dopa residues.

Nicole Balasco,[1] Davide Altamura,[2] Pasqualina Liana Scognamiglio,[3] Teresa Sibillano,[2] Cinzia Giannini,[2] Giancarlo Morelli,[4] Luigi Vitagliano,[5] Antonella Accardo,[4] and Carlo Diaferia[4,]*

[1]Institute of Molecular Biology and Pathology, CNR, Piazzale Aldo Moro 5, 00185-Rome, Italy.

[2]Institute of Crystallography (IC), CNR, Via Amendola 122, 70126-Bari, Italy.

[3]Department of Sciences, University of Basilicata, Via dell'Ateneo Lucano 10, 85100- Potenza, Italy.

[4]Department of Pharmacy and CIRPeB, Research Centre on Bioactive Peptides "Carlo Pedone", University of Naples "Federico II", Via Montesano 49, 80131-Naples, Italy.

[5]Institute of Biostructures and Bioimaging (IBB), CNR, Via Castellino 111, 80131- Naples, Italy.

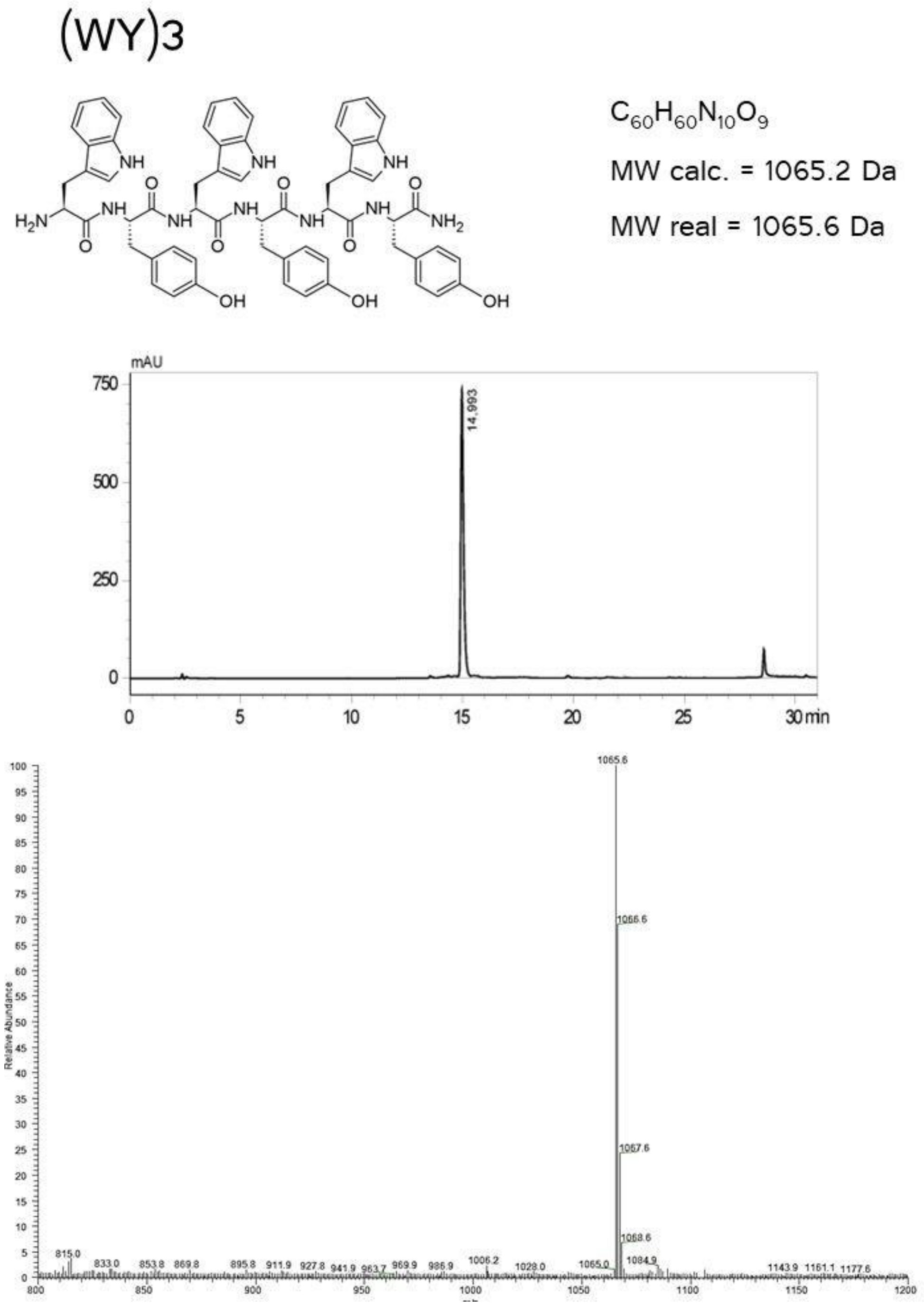

Figure S1: Chemical formula, chromatographic RP-HPLC profile and ESI mass spectrum of (WY)3 peptide.

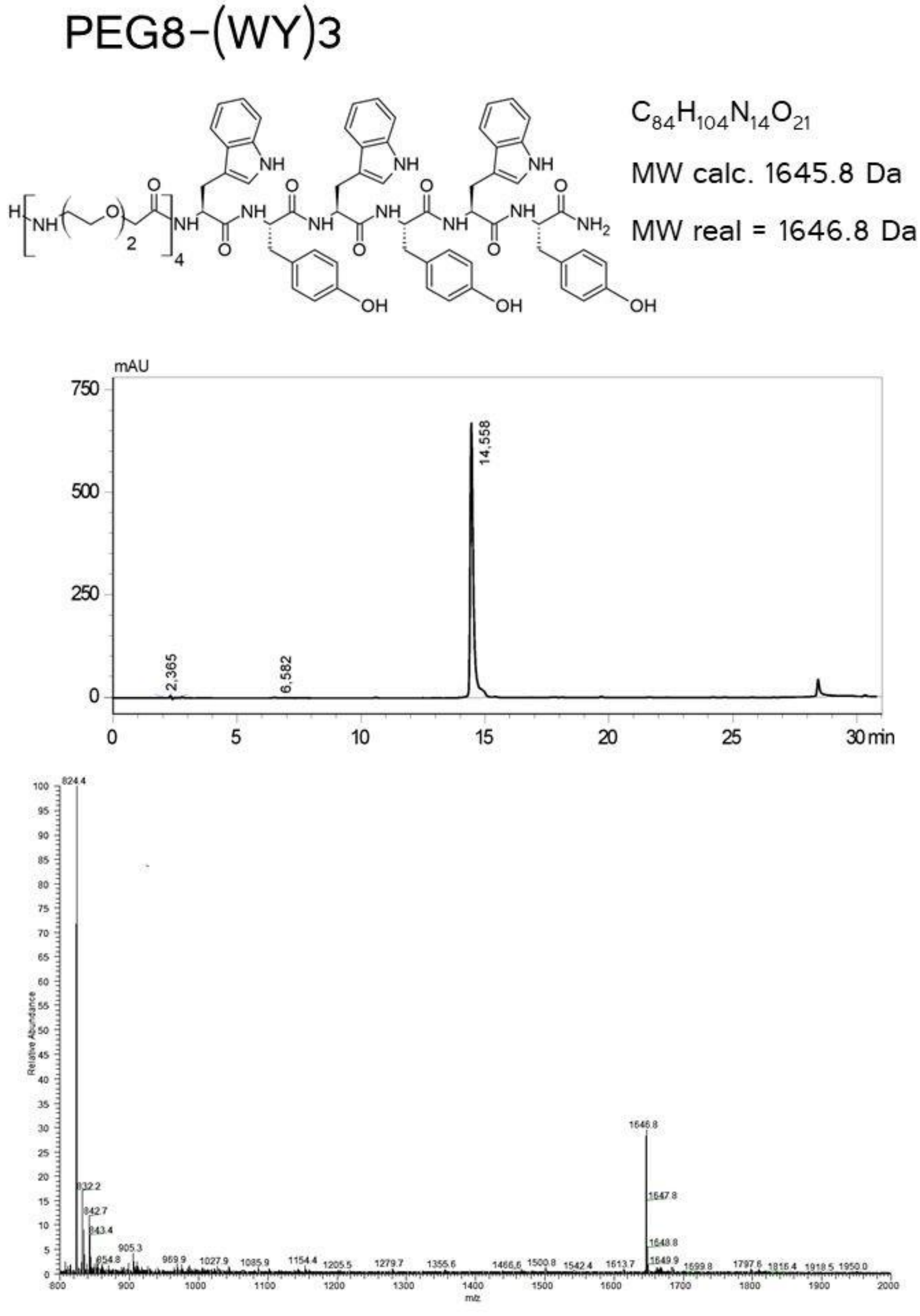

Figure S2: Chemical formula, chromatographic RP-HPLC profile and ESI mass spectrum of PEG8-(WY)3 peptide.

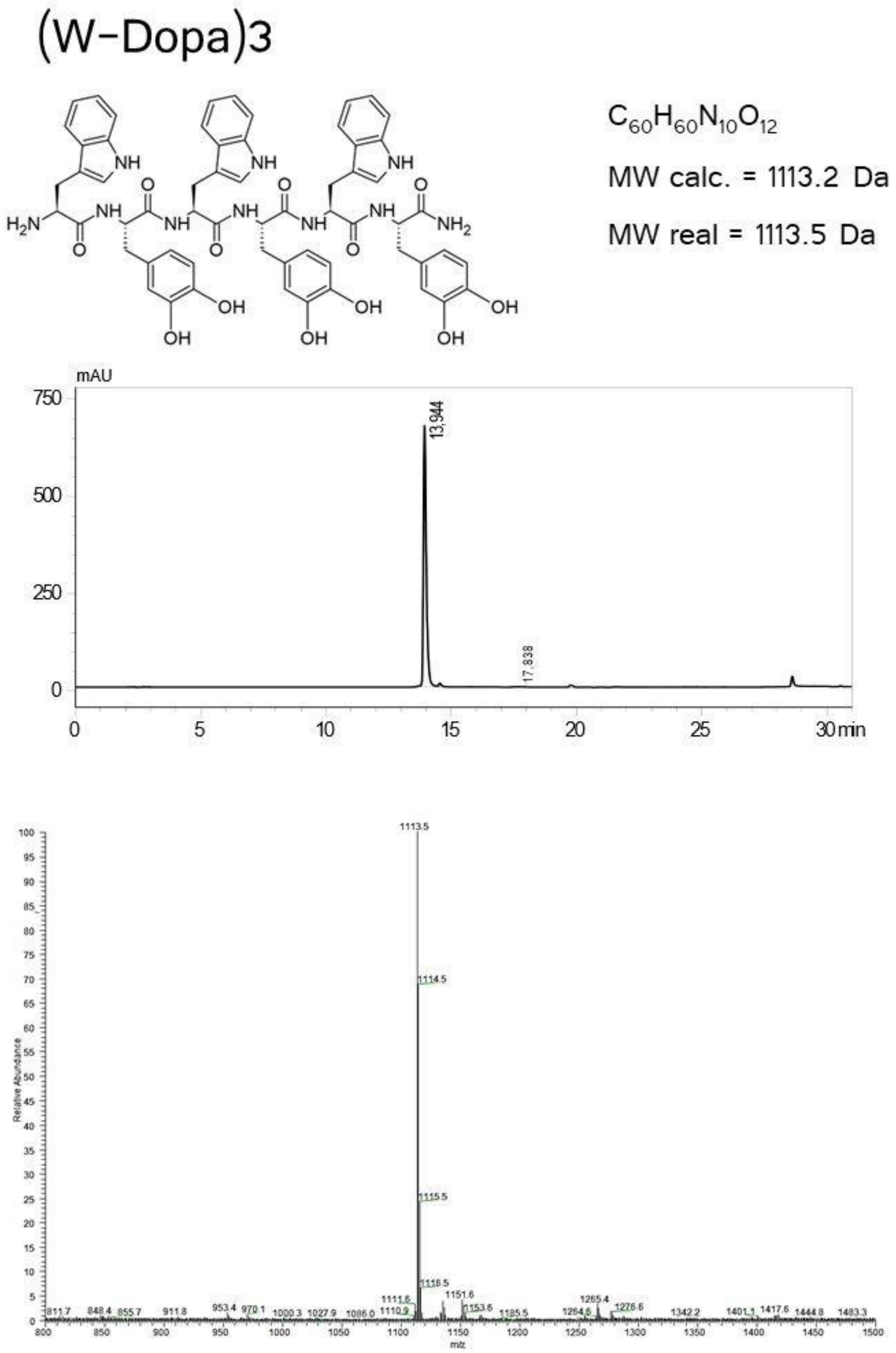

Figure S3: Chemical formula, chromatographic RP-HPLC profile and ESI mass spectrum of (W-Dopa)3 peptide.

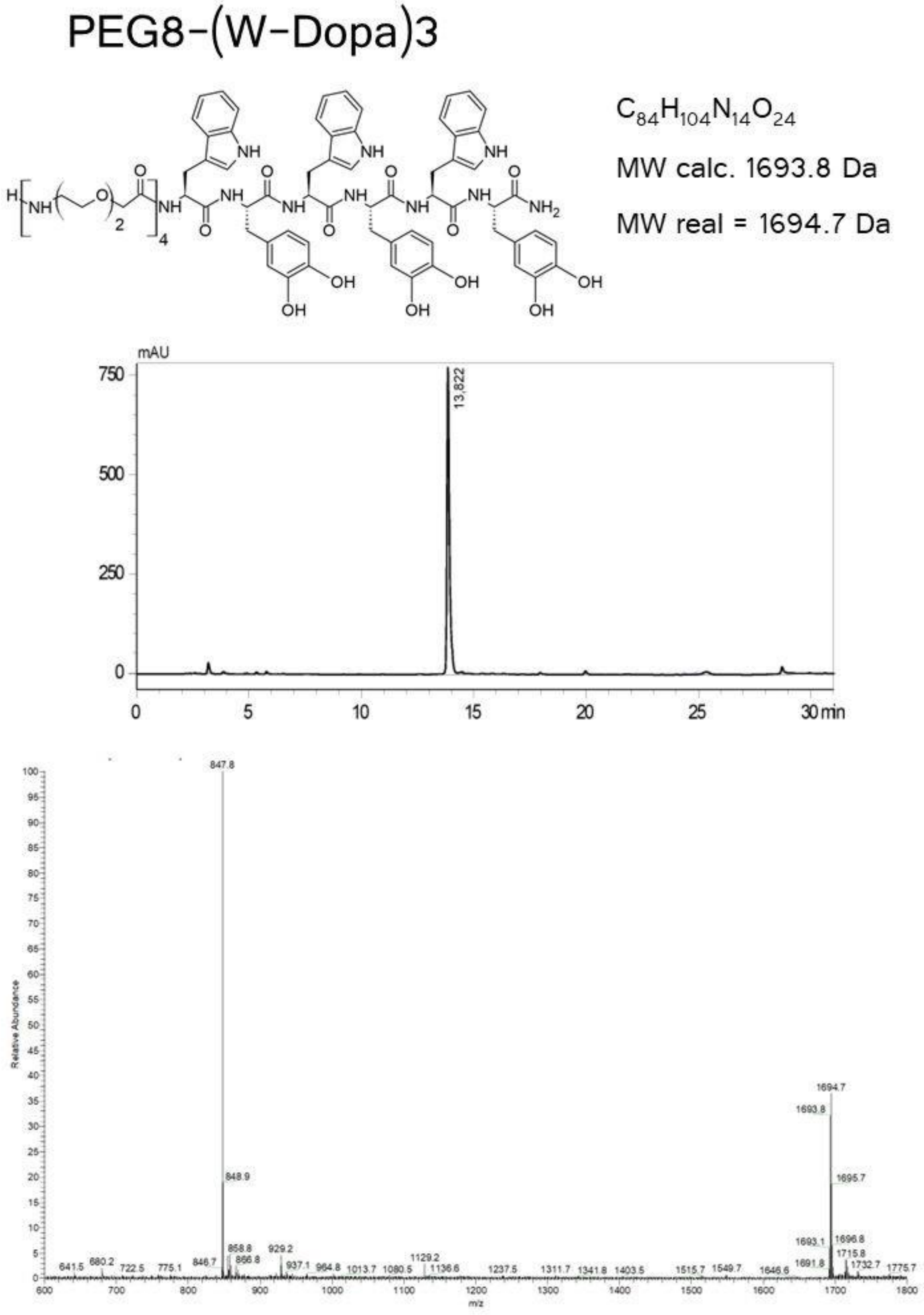

Figure S4: Chemical formula, chromatographic RP-HPLC profile and ESI mass spectrum of PEG8-(W-Dopa)3 peptide.

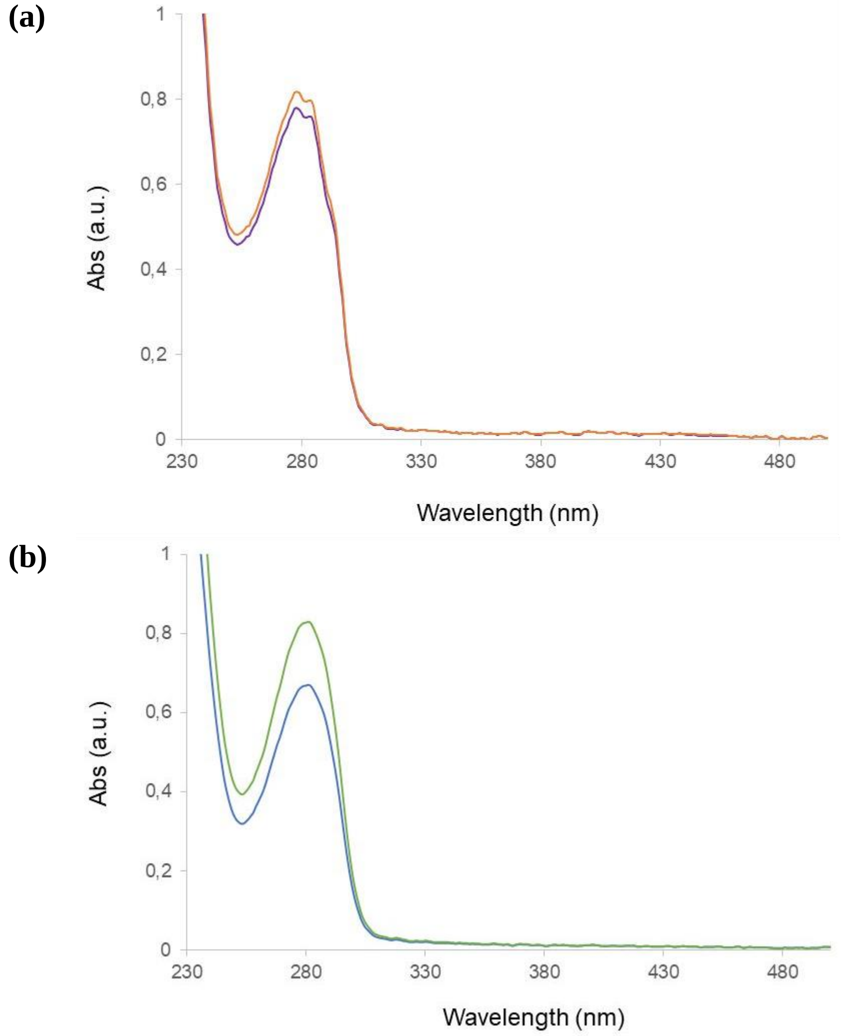

Figure S5: UV-Vis spectra of peptides in their PEGylated and not PEGylated forms: a) (WY)3, [$4.02 \cdot 10^{-4}$ mol/L] in violet and PEG8-(WY)3 [$3.88 \cdot 10^{-4}$ mol/L] in orange; b) (W-Dopa)3 [$3.36 \cdot 10^{-4}$ mol/L] in blue and PEG8-(W-Dopa)3 [$2.66 \cdot 10^{-4}$ mol/L] in green.

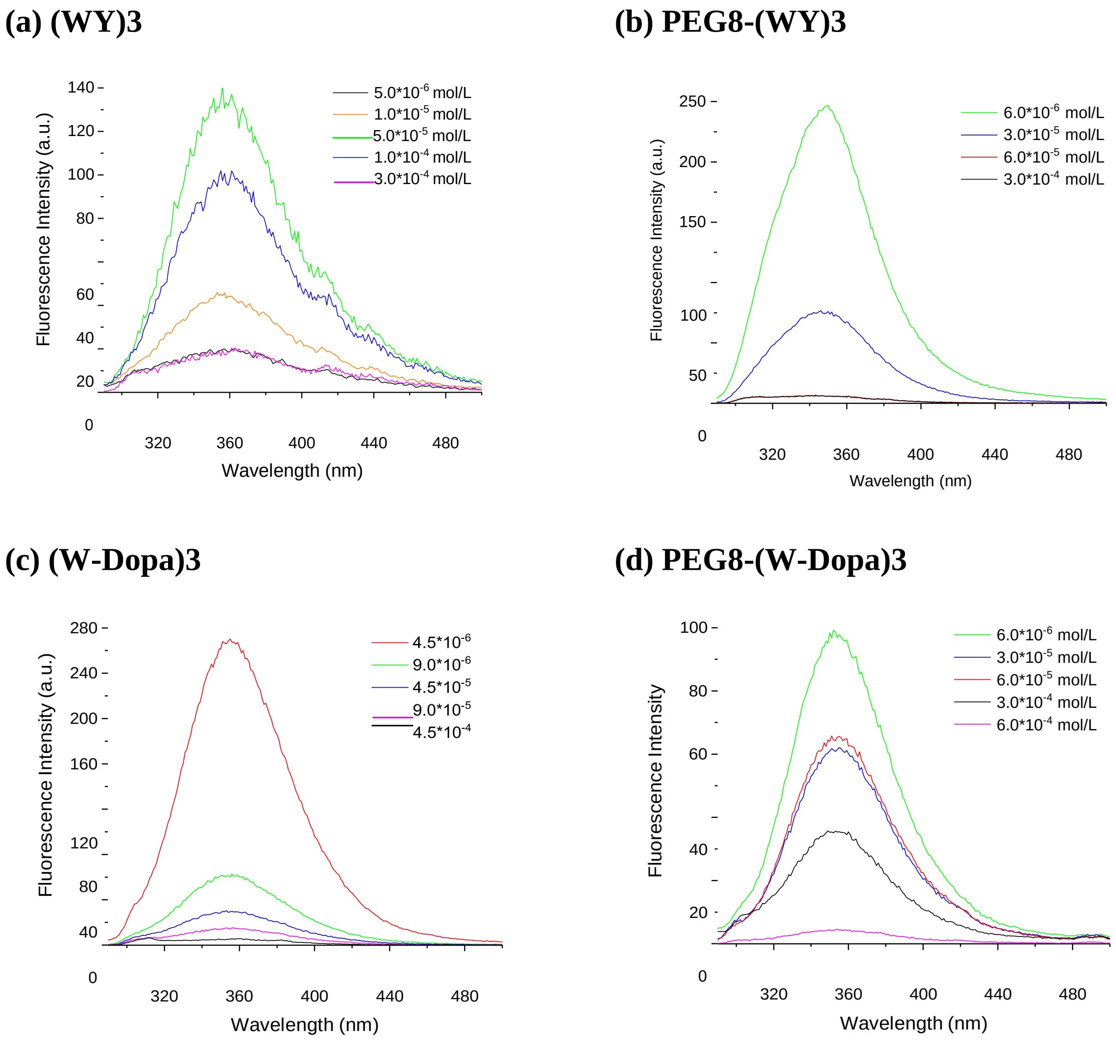

Figure S6: Fluorescence emission spectra of the aromatic peptides at different concentration. Samples have been excited at 280 nm and the emission spectra recorded between 290 and 500 nm.

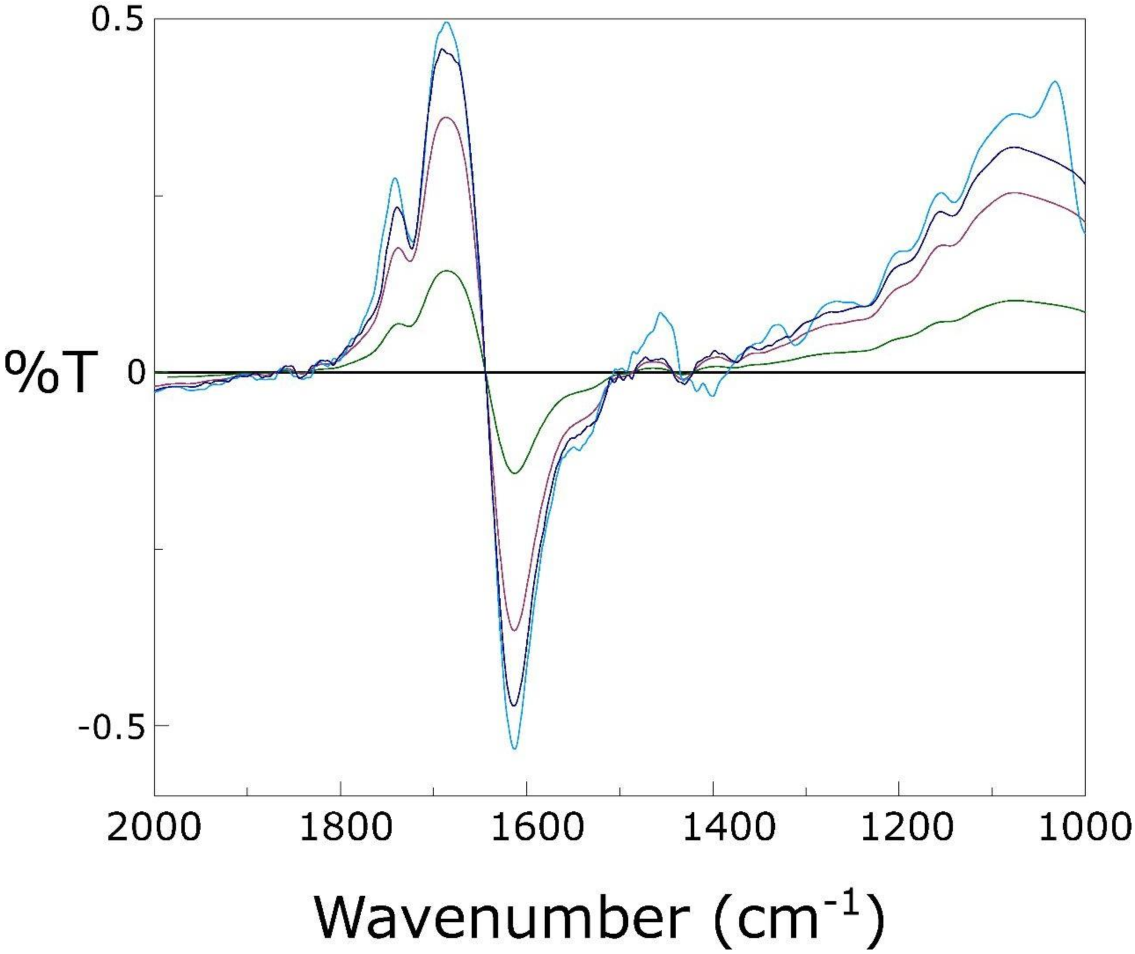

Figure S7: First derivative functions in the 2000-1000 cm^{-1} range. (WY)3 (light blue), PEG8-(WY)3 (green), (W-Dopa)3 (violet) and PEG8-(W-Dopa)3 (blue).

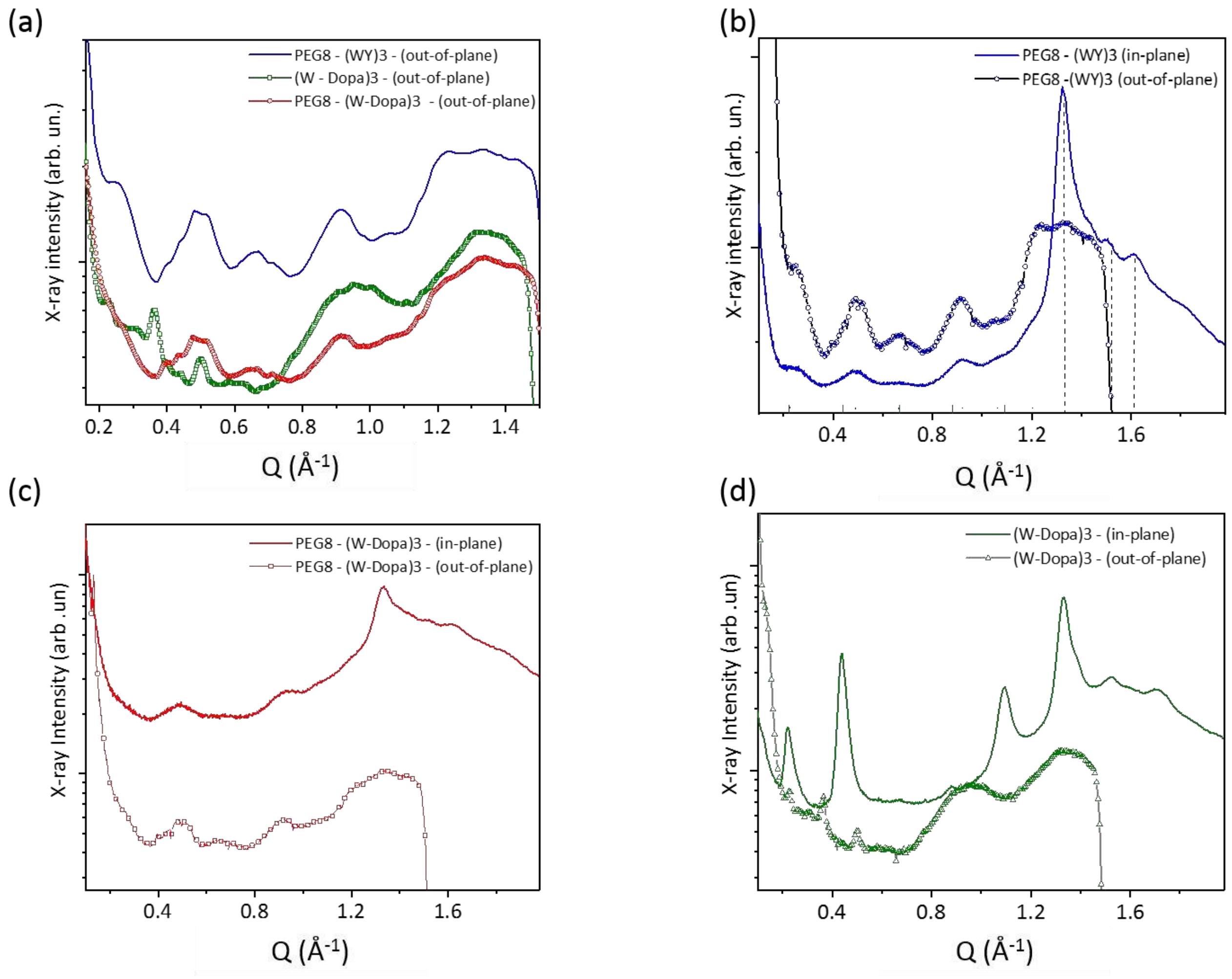

Fig. S8: GIWAXS. 1D linear cuts representing the scattered X-ray intensity along the in-plane and out-of-plane directions.

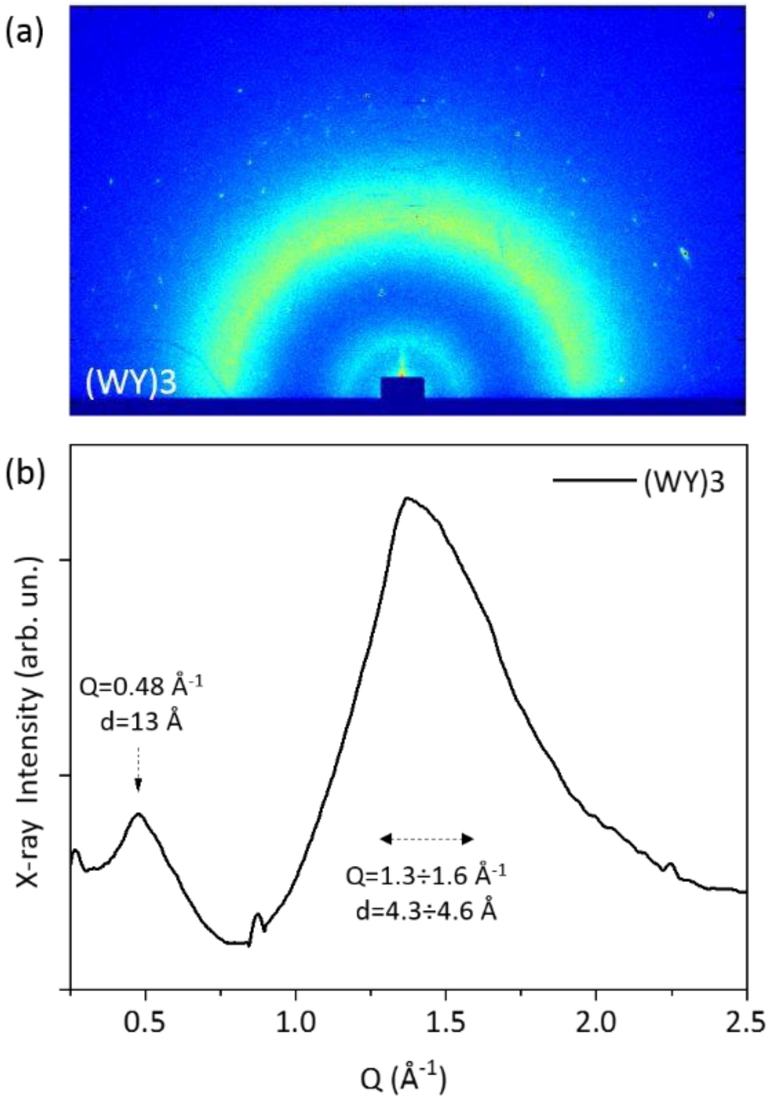

Figure S9: (a) 2D GIWAXS patterns of (WY)3 peptide and (b) the corresponding 1D linear cut representing the scattered X-ray intensity.

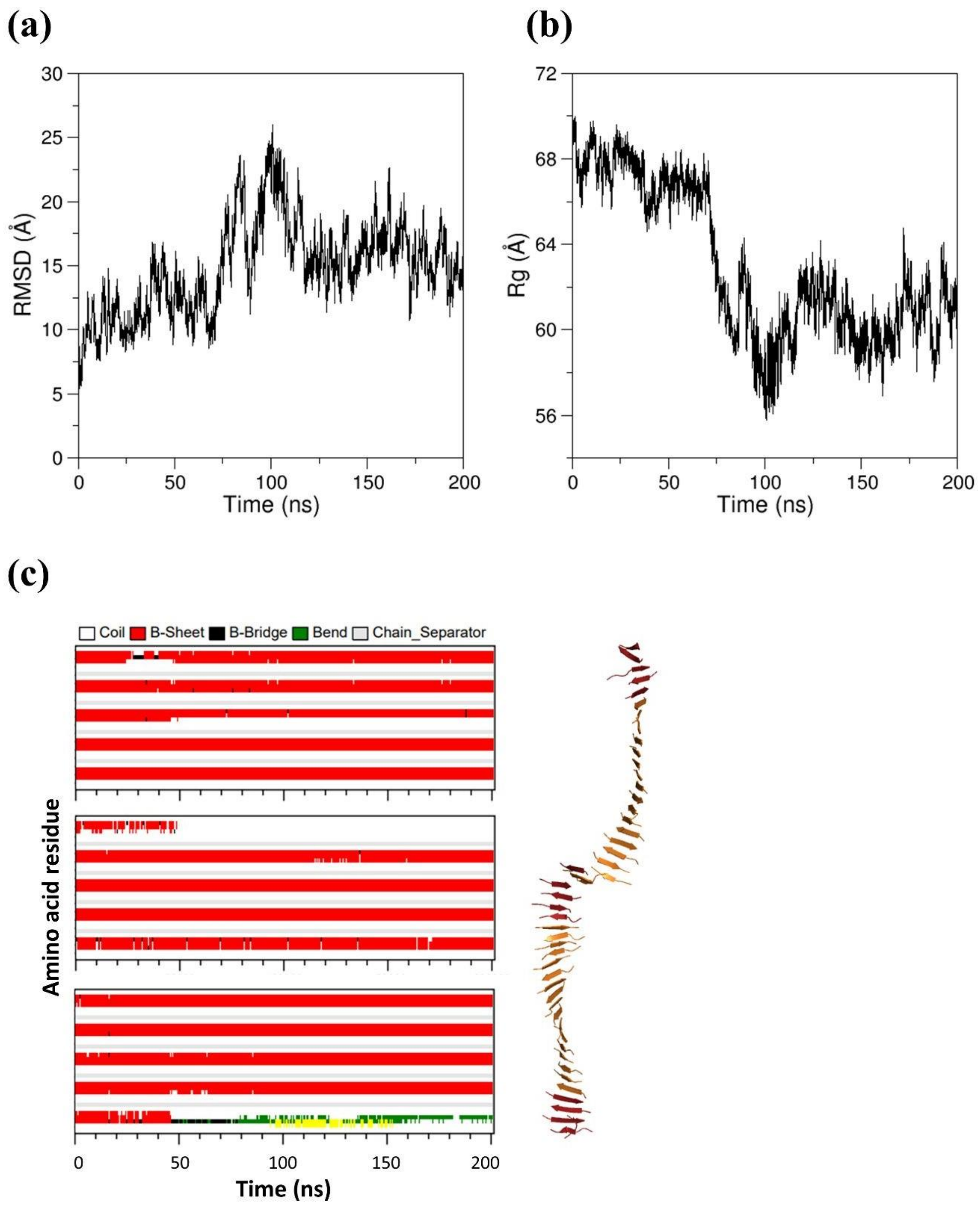

Figure S10: MD parameters evaluated in the simulation of WY_ST50_SH1: (a) RMSD values of trajectory structures against the starting flat model computed on the C$^{\alpha}$ atoms, (b) gyration radius, and (c) secondary structure time evolution. For clarity, the secondary structure is reported only for the residues belonging to the terminal ends and to the central region. The chain separator (light grey) shows the interruption in the polypeptide chain thus allowing to discern the different strands composing the β-sheet. A cartoon representation of the final trajectory structure (time=200 ns) is also reported in panel (c).

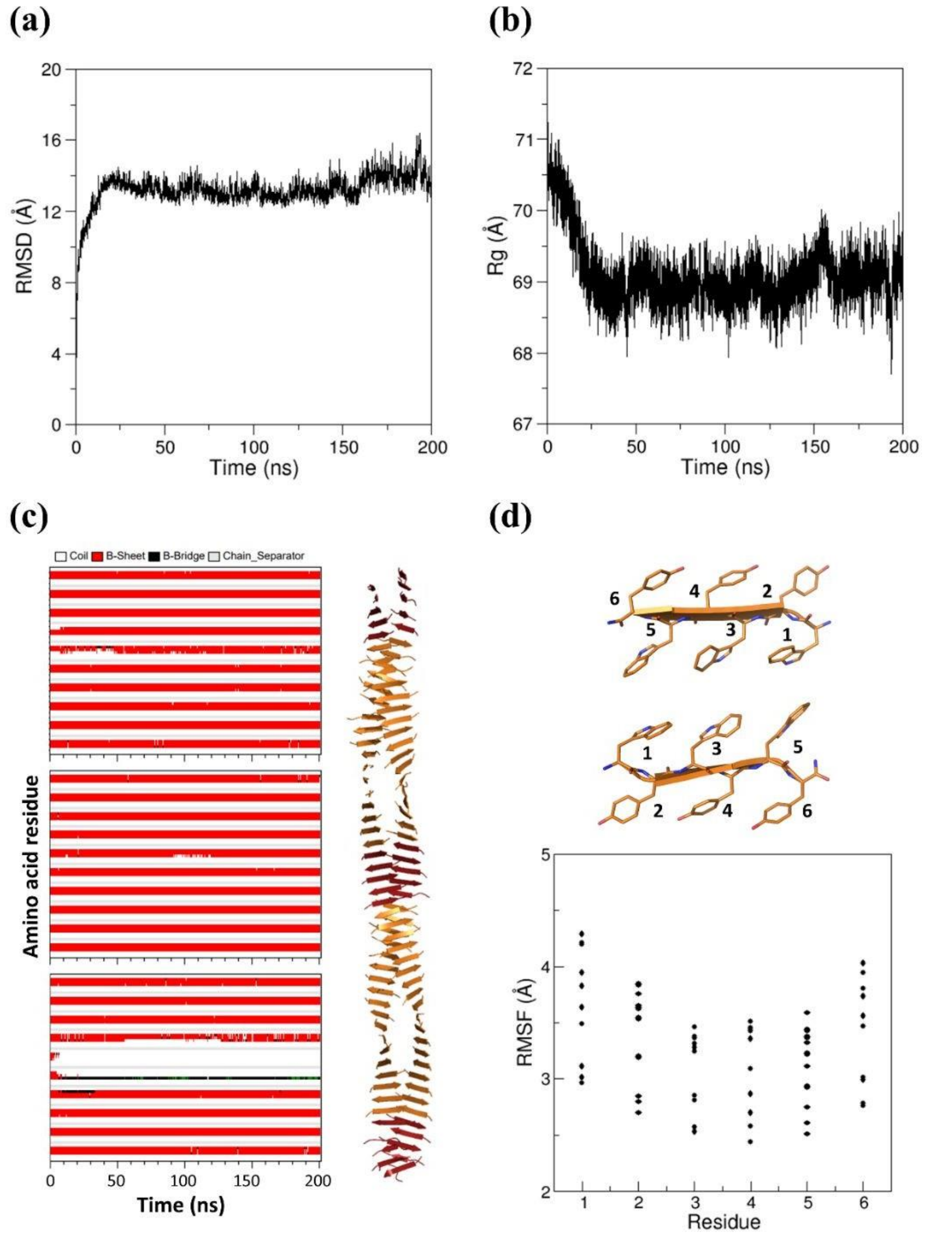

Figure S11: MD parameters evaluated in the simulation of WY_ST50_SH2_WW: (a) RMSD values of trajectory structures against the starting flat model computed on the C^{α} atoms, (b) gyration radius Rg, (c) secondary structure time evolution, and (d) RMSF values computed on the C^{α} atoms of the ten (five *per* sheet) central β-strands in the equilibrated region of the trajectory (50–200 ns). For clarity, the secondary structure is shown only for the residues belonging to the terminal ends and to the central region. The chain separator (light grey) shows the interruption in the polypeptide chain thus allowing to discern the different strands composing the β-sheets. A cartoon representation of the twisted average structure is also reported in panel (c). A stick representation of a couple of facing β-strands is reported in panel (d).

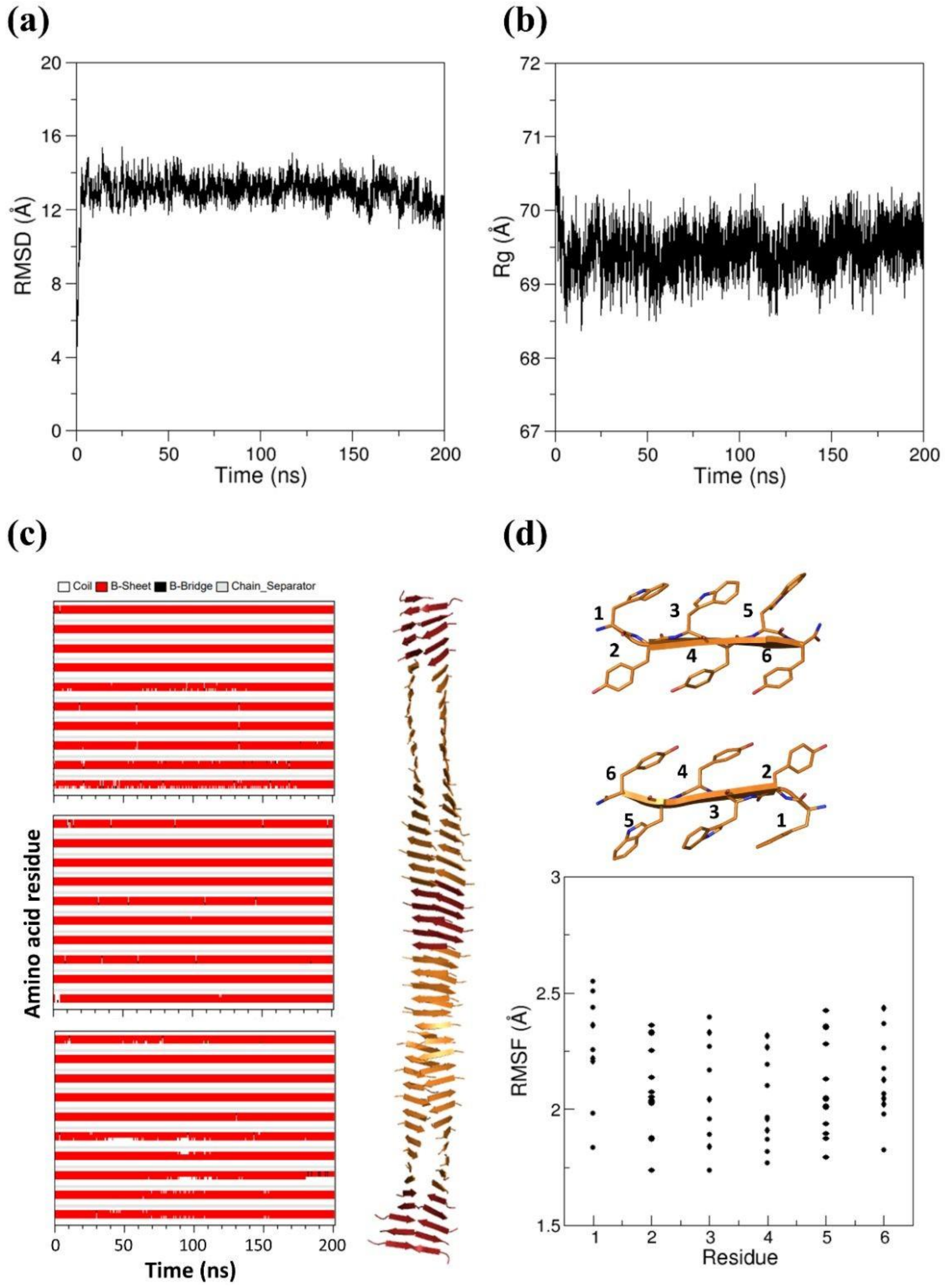

Figure S12: MD parameters evaluated in the simulation of WY_ST50_SH2_WW: (a) RMSD values of trajectory structures against the starting flat model computed on the C^{α} atoms, (b) gyration radius Rg, (c) secondary structure time evolution, and (d) RMSF values computed on the C^{α} atoms of the ten (five *per* sheet) central β-strands in the equilibrated region of the trajectory (50–200 ns). For clarity, the secondary structure is shown only for the residues belonging to the terminal ends and to the central region. The chain separator (light grey) shows the interruption in the polypeptide chain thus allowing to discern the different strands composing the β-sheets. A cartoon representation of the twisted average structure is also reported in panel (c). A stick representation of a couple of facing β-strands is reported in panel (d).

Table S1. MD simulation parameters.

System	Box dimensions (nm^3)	Number of water molecules	Number of Cl^- ions
WY_ST50_SH1	4.63 x 26.03 x 3.47	11652	50
WY_ST50_SH2_WW	5.15 x 26.05 x 4.83	17059	100
WY_ST50_SH2_YY	4.94 x 26.09 x 5.57	19387	100
WY_ST50_SH3	5.21 x 26.09 x 6.29	21666	150

Table S2: Deconvolution percentage functions of secondary structures in amide I region.

Sample	***α-helix (%)***	***β-sheet (%)***	***β-turn (%)***	***Others (%)***
(WY)3	12	63	12	13
PEG8-(WY)3	7	64	19	10
(W-Dopa)3	15	50	17	18
PEG8-(W-Dopa)3	0	52	23	25
